# The trypanosome transcriptome is remodelled during differentiation but displays limited responsiveness within life stages

**DOI:** 10.1186/1471-2164-9-298

**Published:** 2008-06-23

**Authors:** V Lila Koumandou, Senthil Kumar A Natesan, Tatiana Sergeenko, Mark C Field

**Affiliations:** 1 The Molteno Building, Department of Pathology, University of Cambridge, Tennis Court Road, Cambridge, CB2 1QP, UK

## Abstract

**Background:**

Trypanosomatids utilise polycistronic transcription for production of the vast majority of protein-coding mRNAs, which operates in the absence of gene-specific promoters. Resolution of nascent transcripts by polyadenylation and trans-splicing, together with specific rates of mRNA turnover, serve to generate steady state transcript levels that can differ in abundance across several orders of magnitude and can be developmentally regulated. We used a targeted oligonucleotide microarray, representing the strongly developmentally-regulated *T. brucei *membrane trafficking system and ~10% of the *Trypanosoma brucei *genome, to investigate both between-stage, or differentiation-dependent, transcriptome changes and within-stage flexibility in response to various challenges.

**Results:**

6% of the gene cohort are developmentally regulated, including several small GTPases, SNAREs, vesicle coat factors and protein kinases both consistent with and extending previous data. Therefore substantial differentiation-dependent remodeling of the trypanosome transcriptome is associated with membrane transport. Both the microarray and qRT-PCR were then used to analyse transcriptome changes resulting from specific gene over-expression, knockdown, altered culture conditions and chemical stress. Firstly, manipulation of Rab5 expression results in co-ordinate changes to clathrin protein expression levels and endocytotic activity, but no detectable changes to steady-state mRNA levels, which indicates that the effect is mediated post-transcriptionally. Secondly, knockdown of clathrin or the variant surface glycoprotein failed to perturb transcription. Thirdly, exposure to dithiothreitol or tunicamycin revealed no evidence for a classical unfolded protein response, mediated in higher eukaryotes by transcriptional changes. Finally, altered serum levels invoked little transcriptome alteration beyond changes to expression of ESAG6/7, the transferrin receptor.

**Conclusion:**

While trypanosomes regulate mRNA abundance to effect the major changes accompanying differentiation, a given differentiated state appears transcriptionally inflexible. The implications of the absence of a transcriptome response in trypanosomes for both virulence and models of life cycle progression are discussed.

## Background

*Trypanosoma brucei *is the causative agent of sleeping sickness in humans and N'gana in cattle, and has a major economic and morbidity impact across much of Africa [1]. The principal mechanism of immune evasion in the mammalian host is antigenic variation, sequential expression of immunologically distinct variant surface glycoproteins (VSGs) at the cell surface [2], but additional mechanisms, including manipulation of the host immune system and antibody clearance from the surface also participate [3-5]. The full impact of these mechanisms on virulence is not understood at this time. The mammalian part of the life cycle is complex, involving penetration of multiple tissue spaces in the mammalian host, encompassing the bloodstream, lymphatic system and central nervous systems. Despite morphological similarities between mammalian forms, it is unknown if a developmental stimulus is required for tissue tropism or if this results from stochastic events. Further, differentiation to the procyclic insect form on entering the tsetse fly vector is accompanied by massive cellular remodeling, including replacement of the VSG coat with procyclins, a family of acidic glycoproteins, activation of the mitochondrion, changes to cellular and organelle morphology, altered cell cycle checkpoints and attenuation of endocytic activity [6-10]. Multiple stages have been described during fly infection, with clear changes in morphology and surface antigen expression [7,11,12]. How these alterations are controlled at the transcriptional level remains unclear; for example each form could represent a distinct developmental stage, implying stimulus-driven differentiation, or result from transcriptome flexibility, allowing modulation of levels of certain transcripts in response to altered conditions.

Polycistronic transcription in trypanosomes [13] precludes promoter-driven control of transcription, while specific degradation appears to be the major mechanism underpinning regulation of mRNA steady state levels. Microarray hybridization and real time (RT) PCR methods can monitor steady-state RNA levels, and are independent of mechanisms controlling mRNA abundance. An earlier whole genome microarray study [14] identified ~2% of ORFs as developmentally regulated, but several developmentally regulated factors, including the clathrin heavy chain and Rab11 [10,15,16] were not detected in that analysis.

Membrane trafficking is a defining characteristic of eukaryotic cells, playing major roles in nutrient uptake, turnover, signalling, immune defence, apoptosis and many other processes. Mechanisms regulating transport remain only partly understood in any organism, but transcriptional, post-transcriptional and post-translational processes are all implicated, with changes in transcription [17], complex kinase integration [18], and formation of multiple protein-protein interactions [19,20] contributing to control. Further, copious evidence implicates small GTPases in regulating distinct aspects of transport, including Rabs in vesicle fusion [21], ARFs in regulation of membrane coat systems [22] and Rho-related proteins in cytoskeletal interactions [23,24]. Expression profiling for multiple human and mouse tissues indicates correlations between expression of certain Rabs and the SNAREs, adaptors, and other proteins with which they interact [17] and facilitates construction of potential interaction hubs and prediction of novel pathways.

Understanding of membrane transport is comparatively advanced in trypanosomes [25,26], and various cellular changes are associated with life cycle progression [6], most notably an order of magnitude increase in endocytic activity in the mammalian infective bloodstream form (BSF) versus the insect procyclic (PCF) stages [10]. Increased endocytic activity in the BSF is likely related to immune evasion [4,26,27]. By contrast, knowledge of trypanosome signal transduction is poor [28]. Few Ras-like GTPases or their corresponding regulatory factors are present in trypanosomes, heterotrimeric GTPases are absent and there are no obvious receptor-type tyrosine kinases [29-31]. There is a complex predicted trypanosome kinome [31] but limited understanding as to how signaling is mediated *via *these factors [32-34]. There is evidence for phosphatidylinositol-mediated signaling, but this is comparatively unexplored [35], and a large novel family of receptor-coupled adenylate cyclases in kinetoplastids argues for significant environmental sensing *via *novel mechanisms [36,37].

We selected the trypanosome trafficking system as a clear example of a developmentally regulated process, where sufficient data are available for meaningful investigation of transcriptome changes. Using microarray analysis and quantitative RT-PCR, we assessed developmental regulation of trypanosome membrane transport and transcriptional flexibility by targeted genetic manipulation and altered environmental conditions designed to modulate trafficking pathways. From work in other organisms, we anticipated the involvement of protein kinases, small GTPases and, in the case of the unfolded protein response (UPR), a system that responds to increased concentrations of non-native polypeptides within the endoplasmic reticulum, clear evidence for a transcriptional mechanism. Our data indicates developmental remodeling of the transcriptome, but essentially no response to altered conditions.

## Results

### Microarray design

Due to limitations of ORF assignment for specific spots of the previously described whole genome microarray platform [14], we designed a more targeted and fully validated array to address developmental regulation of the trafficking system in *T. brucei*. Major classes of proteins involved in membrane transport include small GTPases, vesicle coats proteins, kinases and phosphatases, motility factors mediating movement of vesicles along cytoskeletal elements, tethering factors for binding of vesicles to target membranes, SNARE proteins, ESCRTs and numerous other molecules involved in protein sorting and membrane deformation. The trypanosome genome was parsed extensively for such factors [38-41] and, based on these and additional searches, a subgenome microarray was constructed. The array also included putative ER chaperones, peptidases bearing an ER signal peptide and potentially involved in degradation within the trafficking system, homologues of kinases implicated in membrane traffic in other systems [18], sphingolipid biosynthesis enzymes with roles in exocytosis of glycosylphosphatidylinositol (GPI)-anchored proteins, as well as trypanosome-specific surface antigens, e.g. BARP, CRAM, and the invariant surface glycoprotein family, or ISGs [42-44]. In addition we also included oligonucleotides against ESAG6 and ESAG7, which encode the trypanosome transferrin receptor, a molecule reported to exhibit expression that is modulated by growth conditions [45].

673 ORFs in the *T. brucei *genome with a potential role in membrane trafficking were identified, representing ~8% of the predicted protein coding potential. Representatives of most components of the major gene families are found in the *T. brucei *genome, but notable exceptions are the absence of several coat systems [46], a relatively small number of GTPases and associated factors [30], and the absence of BAR and SH2 domains; BAR domains are used extensively for membrane deformation in higher eukaryotes [47], while the absence of SH2 domains may indicate limited or unconventional signalling through protein tyrosine kinase pathways [40,48]. Apart from clustering of multicopy genes, there is no location bias for a specific chromosome or region within a chromosome for these ORFs [see Additional file 1]. An additional 123 ORFs were also included as a reference. The first array (v1.0), used for comparison of life stages and analysis of Rab5 isoform over-expression, comprised 694 oligonucleotides, 56 of which targeted reference ORFs. A modified array (v1.1), used for all other analyses, included an additional 49 oligonucleotides. The vast majority of oligonucelotides are gene-specific but some are expected to hybridise to multiple transcripts from multicopy genes [see Additional file 2].

### Overview of differential expression between trypanosome life-stages

**Figure 1:**
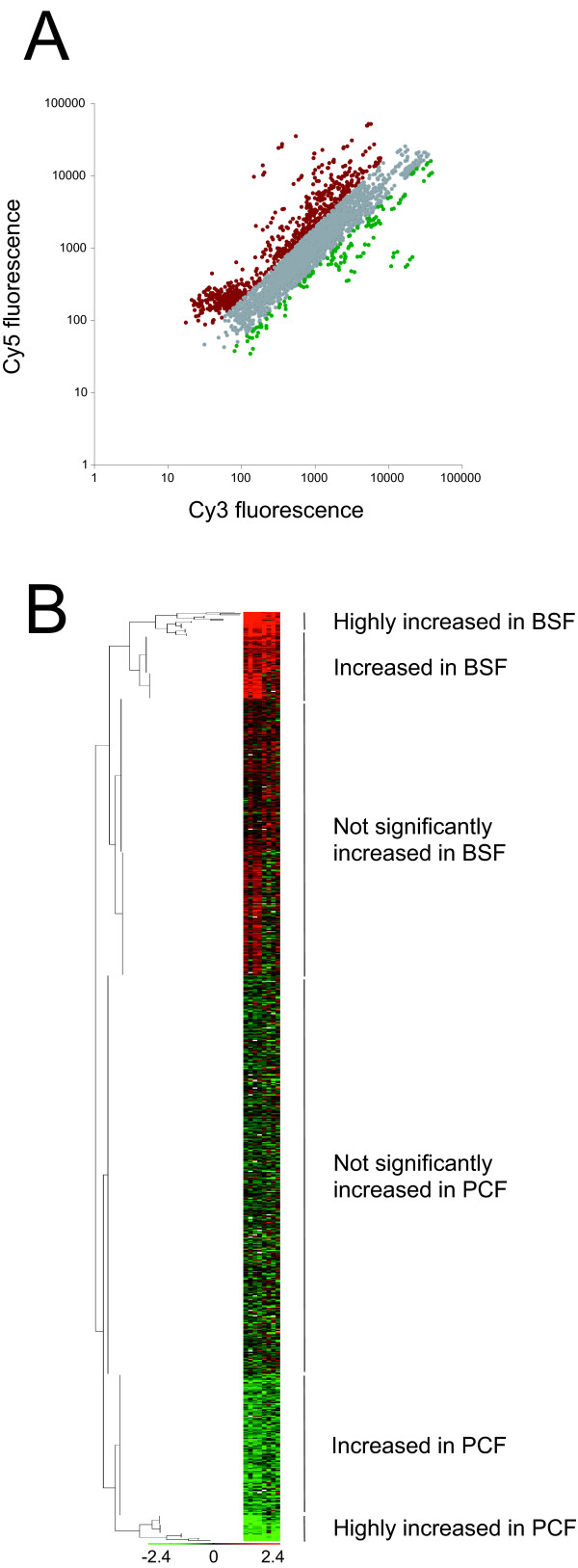
**Overview of developmental transcriptome changes in the membrane trafficking system of trypanosomes**. Panel A: Scatter plot of raw data for all 3600 spots on a representative microarray used for developmental expression experiments. Cy5 fluorescence is plotted on the Y-axis (bloodstream, BSF) and Cy3 fluorescence on the X-axis (procyclic, PCF). Spots with a BSF/PCF ratio above two are highlighted in red, while spots with a PCF/BSF ratio above two are highlighted in green. Panel B: Clustering of the data for eight microarray experiments comparing BSF to PCF, representing four biological replicates plus relevant dye-swaps. The scale indicates the colour scheme for the z-score of the data, i.e. how far and in what direction, the ratio for each spot deviates from the mean for each array, expressed in units of standard deviation; bright red indicates significant upregulation in BSF, bright green indicates significant upregulation in PCF, dark colours or black indicate no differential expression between the two developmental stages. For each target gene, the four replicate spots on the array were averaged. White indicates data points rejected because of too much variability in the replicate spots. A small but significant number of ORFs exhibit strong developmental regulation.

We extracted RNA from exponentially growing cultures of *T. brucei *bloodstream and procyclic forms, labelled and competitively hybridised the cDNA to the microarray. As expected, the majority of genes were equivalently expressed in the two life stages, but a small cohort did present significant differential expression (Figure 1). The heat-map (Figure 1B) indicates that the data for the most significantly developmentally regulated genes are highly reproducible across all replicates (8 arrays, 4 replicate spots per array).

To identify which mRNAs are differentially expressed, limma and fspma packages were used to interrogate the data [49-51]. For comparison with previous work [14], ratios above 2.5 (+/- 1.325, log_2 _scale) were considered highly significant. Using this threshold, 8% of the genes represented on the array are upregulated in BSF and 5% in PCF, but excluding the reference set these numbers reduce to 4% in BSF and 2% in PCF. Using a whole genome microarray, Brems and cowrokers found only 2% of trypanosome genes were developmentally regulated, and while different pre-processing steps, in both mRNA preparation and data analysis, make direct comparison difficult, the present data suggest that the trafficking cohort likely exhibits bias in developmental regulation. There is also greater upregulation in BSFs compared to PCFs, consistent with increased activity in the trafficking system in bloodstream stages [10]. Brems *et al*. found 80 genes upregulated in PCF and 34 in BSF while, using the same threshold, the present analysis identifies 16 upregulated in PCF and 34 in BSF, from a much smaller cohort. These data suggest that significant transcriptional level remodelling of the membrane trafficking system accompanies life-cycle progression in trypanosomes.

Strongly developmentally regulated genes include several reference ORFs with known regulated expression, i.e mRNAs for procyclin, *trans*-sialidase, CRAM, CAP17, and CAP5.5 are increased in PCF, while ESAG6, ESAG7, ISG65, ISG75, GPI-PLC, and CAP15 are upregulated in BSF (Table 1). These data are fully consistent with previous work and support the validity of the microarray approach for identifying previously uncharacterised developmentally expressed genes. Rab11, also developmentally regulated [15], gave an expression ratio below 2.5 by both limma and fspma analysis. Hence, analysis using a more relaxed threshold to identify developmentally regulated mRNAs was also used. Table 1 lists all ORFs which either gave a ratio above 2.0 (+/- 1.00, log_2 _scale) by limma or fspma analysis, or which had a B-statistic value greater than 1.0 in limma, indicating statistical significance in differential expression, despite a low relative expression ratio. Excluding the reference set, 101 genes with a possible role in membrane transport are differentially expressed in *T. brucei*; 64 upregulated in BSF, 37 in PCF, i.e. 8% and 5% respectively.

**Table 1:** Transcripts differentially expressed between the procyclic and bloodstream forms of *T. brucei*.

**A**					
**Upregulated in the procyclic form**					
		**PCF**/BSF ratio			
			
Accession	Name	limma	fspma	RT-PCR	Classification

*Tb10.6k15.0030	**procyclin**	**22.3**	34.6		Cell surface
*Tb927.6.450	**procyclin**	**21.9**	17.0		Cell surface
Tb927.7.6850	***trans*-sialidase**	**5.0**	4.7		Cell surface
Tb10.6k15.3510	**CRAM**	**3.8**	3.8		Cell surface
*Tb09.244.2460	BARP	**1.9**	1.9	1.6	Cell surface

Tb11.01.8120	hypothetical ER chaperone	**3.0**	3.0	2.8	Chaperones
*Tb927.6.3740	**Hsp70 mitochondrial**	**2.3**	4.2		Chaperones
*Tb10.70.0280	**Hsp60 chaperonin mitochondrial**	2.0	2.1		Chaperones
Tb11.01.3110	**Hsc70**	1.6	2.8		Chaperones

*Tb927.8.1610	**Gp63 (TbMSP-B)**	**3.3**	5.6	3.8	Proteases
Tb11.02.1480	mitochondrial processing peptidase	**5.6**	5.1	5.6	Proteases
Tb09.211.4760	MCA5 metacaspase	2.2	2.1	1.3	Proteases

Tb10.61.0870	v-SNARE	**2.9**	2.8		SNAREs

Tb927.4.2020	AP3 mu	**2.1**	2.2		Vesicle coat
Tb927.4.4350	emp24	**2.1**	1.9	1.0	Vesicle coat

Tb927.3.4020	PI4K alpha	**2.2**	2.1	1.7	PI kinases
Tb10.6k15.2060	PI3K (FRAP1)	**2.0**	2.0		PI kinases

Tb11.01.7800	NDPK kinase (NME6)	2.7	4.4		Other kinases
Tb11.03.0090	ribokinase (RBSK)	**2.7**	3.4		Other kinases
Tb927.3.3190	NEK kinase (NEK2/6/7)	**2.5**	2.9		Other kinases
Tb10.389.0330	UGP2	**2.4**	2.2		Other kinases
Tb927.6.710	DPCK kinase	**2.0**	2.0		Other kinases
*Tb09.211.3540	glk1 (CARKL)	1.4	2.9		Other kinases
*Tb09.160.4560	AK	1.4	2.8		Other kinases
*Tb927.1.720	PGKA (PGK1)	**1.9**	2.4		Other kinases

Tb11.02.0580	Vps46	**2.4**	2.8		ESCRT

Tb927.2.4210	**p60 glycosomal protein**	**9.9**	7.4		Other trafficking
Tb11.01.7880	**CAP17**	**4.5**	3.3		Other trafficking
Tb927.4.3950	**CAP5.5**	4.1	3.2	8.0	Other trafficking
Tb927.7.2640	Sec34-like	**8.3**	7.9	3.7	Other trafficking
Tb11.01.2540	VHS domain-containing protein	2.6	2.8		Other trafficking
Tb11.47.0022	signal-peptide-containing protein	**3.1**	2.7		Other trafficking
Tb10.406.0240	dual specificity protein phosphatase	**2.1**	2.6		Other trafficking
Tb927.1.3110	Sec17/SNAP	1.8	2.5		Other trafficking
Tb09.160.3240	C2 domain-containing protein	**2.0**	1.9		Other trafficking
Tb927.7.3550	C2 domain-containing protein	**1.8**	2.5		Other trafficking
Tb10.100.0130	peroxin 14	2.1	1.9		Other trafficking

Tb09.211.2150	PABP2	2.7	2.4		Miscellaneous
Tb927.8.7120	farnesyltransferase	**2.6**	2.5		Miscellaneous
*Tb10.406.0330	**histone H2B**	2.5	2.8	1.0	Miscellaneous
*Tb927.3.1370	40S ribosomal protein S25	**2.4**	3.2	1.5	Miscellaneous
Tb11.02.5250	histone H2B, variant	2.3	1.7	0.9	Miscellaneous
*Tb927.1.2430	**histone H3**	2.2	2.9	1.1	Miscellaneous
*Tb927.1.2400	**alpha tubulin**	1.7	3.0	0.8	Miscellaneous
Tb927.8.4010	**fla1**	**1.8**	2.2		Miscellaneous
Tb11.01.1650	SRP54-like	**2.0**	2.3		Miscellaneous
Tb927.3.3020	actin-like	1.5	2.7		Miscellaneous

**B**					

**Upregulated in the bloodstream form**					
		**BSF**/PCF ratio			
			
Accession	Name	limma	fspma	RT-PCR	Classification

*H25N7.15	**ESAG6**	**52.0**	27.7		Cell surface
*N19B2.155	**ESAG7**	**27.7**	22.8		Cell surface
*Tb927.5.1410	**ISG65**	**14.8**	9.6		Cell surface
*Tb927.2.3270	**ISG65**	**7.2**	4.8		Cell surface
*Tb927.2.3280	**ISG65**	**7.0**	5.5		Cell surface
*Tb927.2.3320	**ISG65**	**6.4**	3.9		Cell surface
*Tb927.5.390	**ISG75**	**5.8**	8.2		Cell surface
Tb11.47.0001	**ISG65**	2.4	1.7		Cell surface
*Tb09.244.2430	**BARP**	2.4	2.4		Cell surface
*Tb927.5.360	**ISG75**	**2.3**	2.4		Cell surface

Tb927.7.5790	PDI/ERp44	2.5	2.8		Chaperones
Tb11.01.2640	hypothetical ER chaperone	2.3	2.2	1.0	Chaperones

Tb927.3.3450	ARL3A	**7.0**	5.9	7.8	small GTPases
*Tb05.5K5.230	Rab1b	**7.0**	8.2		small GTPases
Tb927.4.2380	HSR1-related GTP-binding protein	**3.9**	3.4	5.2	small GTPases
*Tb927.5.4590	Rab1b	**3.8**	5.7		small GTPases
*Tb09.244.2070	Rab1b	3.5	2.4		small GTPases
Tb927.8.8140	TbRX3	2.8	2.4		small GTPases
Tb10.70.0590	TbRHP	**2.3**	2.1		small GTPases
Tb11.01.6670	RabX3	2.3	2.1	4.8	small GTPases
Tb927.8.4330	Rab11a	2.3	2.0	1.9	small GTPases
*Tb09.211.4460	TbARF1	**2.0**	2.0		small GTPases

Tb10.70.5290	**Gp63 (TbMSP-C)**	**4.7**	3.3	6.2	Proteases
Tb927.3.4230	Ser peptidase (sybtilisin-like)	**5.6**	7.5	3.3	Proteases
Tb09.211.0680	CAAX prenyl protease 1	**2.5**	2.3	2.7	Proteases
Tb11.02.1280	Ser peptidase (sybtilisin-like)	2.5	2.2	1.1	Proteases
*Tb927.6.930	**MCA3 metacaspase**	**2.3**	1.9	1.8	Proteases
Tb927.7.190	OPA Thimet oligopeptidase	2.1	2.0	1.2	Proteases

Tb09.160.2420	syntaxin	**3.6**	2.6		SNAREs
Tb927.7.6440	v-SNARE	**2.9**	3.5		SNAREs
Tb927.3.3720	t-SNARE (Bet1)	**2.5**	2.7		SNAREs
Tb09.211.3920	t-SNARE	2.5	1.8	1.8	SNAREs
Tb10.70.7410	VAMP (synaptobrevin)	**1.8**	2.5		SNAREs

Tb10.389.0480	Vps16	1.9	2.8		Tethers

Tb10.61.1910	CLL	**3.1**	2.5	4.2	Vesicle coat
Tb927.3.4000	AP1 sigma	**6.0**	7.0		Vesicle coat
Tb11.01.6880	emp24	2.1	2.2	1.7	Vesicle coat
*Tb11.50.0006	epsin-like protein	**2.0**	2.1	2.5	Vesicle coat

Tb11.01.6980	PI3K (FYVE domain)	7.5	10.3		PI kinases
Tb927.8.7110	Ser/Thr kinase NEK7-like (PH domain)	**2.6**	2.1		PI kinases
Tb10.70.2440	PIK5KII	2.1	2.6		PI kinases
*Tb927.4.5310	Ser/Thr kinase A (PH domain)	**1.9**	1.7		PI kinases

*Tb10.70.5800	**hexokinase (GCK/HK)**	3.8	3.8		Other kinases
Tb927.6.1780	MAP kinase (MAPK8/9)	**2.9**	2.3		Other kinases
Tb927.4.3770	calcium-dependent protein kinase (CAMK4)	2.5	3.2		Other kinases
Tb10.61.2680	**pyruvate kinase (PKLR)**	2.2	1.8		Other kinases
Tb10.61.2490	MAP kinase kinase kinase (MAP3K8)	2.2	2.0		Other kinases

Tb10.70.1130	two UIM domain-containing protein	**8.8**	5.9		Ubiquitin sorting
Tb11.46.0014	Doa4 (Ubiquitin C-term hydrolase)	**2.7**	2.9		Ubiquitin sorting
Tb09.211.3610	UBA2/E1	**2.3**	2.4	2.5	Ubiquitin sorting
Tb927.6.2370	Ubiquitin-protein ligase	2.2	2.2		Ubiquitin sorting
Tb927.4.2710	E2	1.6	2.5	1.6	Ubiquitin sorting

Tb927.2.6000	**GPI-PLC**	**8.7**	8.0		Other trafficking
Tb11.01.3805	**CAP15**	**4.3**	4.8		Other trafficking
Tb09.211.4240	sorting nexin 2 (PX domain)	**3.4**	4.8		Other trafficking
Tb10.70.4620	gamma SNAP	**2.7**	2.5		Other trafficking
Tb927.4.2070	giantin	**3.1**	1.8		Other trafficking
Tb927.6.3500	RME-8 endosome trafficking	**2.8**	2.2	2.3	Other trafficking
Tb11.01.4460	scavenger receptor	3.0	2.8		Other trafficking
Tb927.5.4520	Uso1p-like (spectrin repeat)	2.7	1.8		Other trafficking
Tb927.5.3220	Sec11	2.4	3.4		Other trafficking
Tb11.01.6260	Sec63	2.3	2.3		Other trafficking
Tb927.1.1560	NSF	2.2	1.7		Other trafficking
Tb10.70.5100	lysosomal alpha-mannosidase	2.2	1.8		Other trafficking
Tb11.02.1370	katanin/Ser peptidase	**1.7**	1.7		Other trafficking
Tb927.4.2080	C2 domain-containing protein	**1.9**	1.8		Other trafficking

*Tb927.5.420	ISG65-like	3.5	5.1		Miscellaneous
Tb927.5.310	ISG-like	**3.3**	3.2		Miscellaneous
*Tb927.8.4060	fla2 (fla1-like)	3.5	2.6		Miscellaneous
Tb10.61.1090	H3 variant	2.3	2.9	1.8	Miscellaneous
Tb10.406.0320	ARPC5	2.2	2.5		Miscellaneous
Tb927.5.630	ISG65-like	2.2	2.4		Miscellaneous
Tb927.5.430	ISG65-like	2.2	2.1		Miscellaneous

ORFs represented by oligonucleotides on the array which gave a PCF to BSF expression ratio (panel A) or BSF to PCF expression (panel B) greater than two by limma or fpmsa analysis, or which had a value of over one for the B statistic in limma are shown. Limma ratios which correspond to a B value of >1 are given in bold. For candidates validated by qRT-PCR, the resulting qRT-PCR ratio is also given. Ratios below two may not be significant. The names of well-characterised genes which can be used as controls to validate the array results are in bold. Asterisk indicates oligonucleotides that hybridise with mRNA derived from more than one ORF [see Additional file 2].

25% of the most significantly developmentally regulated genes were validated by qRT-PCR analysis, using the same mRNA samples as used for the microarray analysis. The vast majority of ORFs tested exhibited congruence between qRT-PCR and microarray analysis, i.e. differential regulation in the same direction (BSF or PCF, Table 1). Significantly, all ORFs with a ratio above 2.5 on the array also gave a significant ratio (i.e. above 2) in qRT-PCR. Two genes with known developmental regulation, the clathrin heavy chain [10,16] and TbMSP-A [52], did not demonstrate significant differential regulation by microarray analysis, but highly significant ratios were obtained by qRT-PCR (data not shown). Various factors may be responsible for this array artefact, which were not pursued further; however these data specifically highlight examples of false-negative data in microarray analysis.

### Differential expression of trafficking factors in trypanosomes

**Figure 2:**
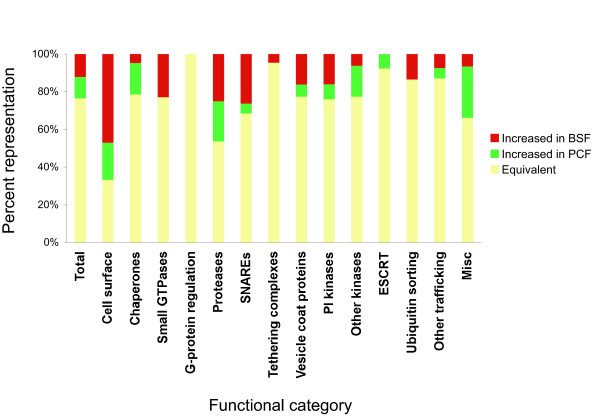
**Significantly developmentally expressed trypanosome genes grouped by functional class**. Genes represented by array oligonucleotides were grouped by function, based on sequence similarity to annotated sequences, domain annotation, GO terms and additional criteria. The proportion in each functional class significantly upregulated in BSF are shown in red and in PCF in green. The remaining fraction of genes in each class are shown in grey. The first bar illustrates the proportion of developmentally regulated transcripts in the entire studied gene cohort. For raw data [see Additional file 6].

Considering the differentially expressed genes by functional class indicates that a broad remodelling accompanies differentiation, i.e. developmental changes are not restricted to specific protein classes or pathways. Specifically, members of the chaperones, some proteases and non-PI kinases are upregulated in PCF, while the small GTPases, additional proteases, SNARE proteins and PI kinases represent the major factors upregulated in BSFs (Figure 2, Additional file 6). An increased requirement for chaperones may be due to the greater heterogeneity of the procyclic cell surface compared to the bloodstream form, the latter being dominated by VSG. A differential requirement for secretory pathway proteases is consistent with distinct roles for these molecules between the two life stages, while upregulation of both small GTPases and SNARE proteins in BSFs is broadly consistent with increased activity within the trafficking system in the mammalian stage.

#### Specificity and control factors

**Figure 3:**
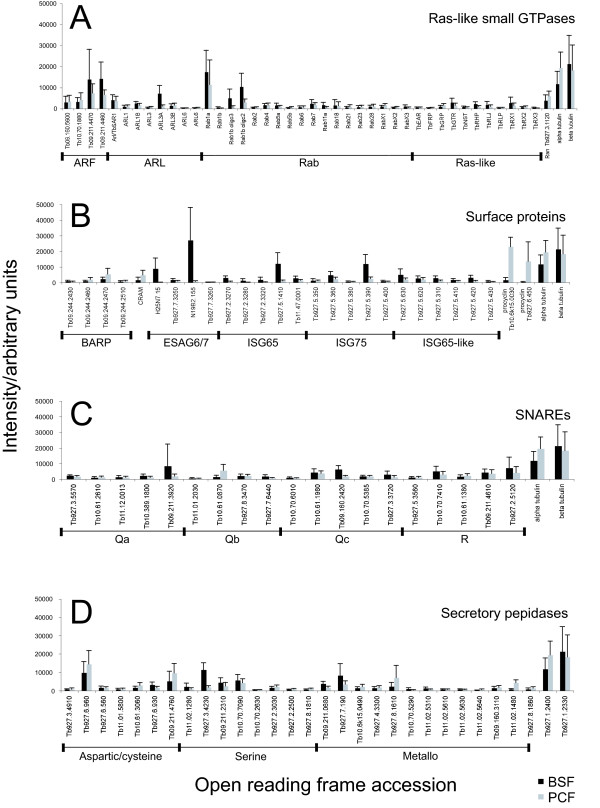
**Relative steady state mRNA levels of selected trypanosome genes**. Fluorescence intensity data for eight microarray experiments comparing BSF to PCF, representing four biological replicates plus dye-swaps, was normalised to the total signal intensity of each slide, averaged and plotted as bar-graphs against ORF designation or accession number. Error bars indicate the standard deviation, dark bars are BSF and light bars PCF. Panel A: Small GTPases. ARL and Rab annotations are based on [39], Ras-like annotations based on [29] and additional data at GeneDB. Panel B: Cell surface proteins. Panel C: SNARE family proteins. Annotations based on domain architecture and similarity to sequences described for *L. major *[59]. Panel D: Putative secretory pathway proteases. ORFs were included for predicted proteases that bear an ER targeting sequence and/or a predicted *trans*-membrane domain. In each cohort, a limited number of transcripts are highly expressed.

The most abundantly expressed small GTPases at the mRNA level are Ran, ARL3A, both Rab1A and Rab1B, and several members of the Arf subfamily (Figure 3). Ran is a very abundant protein in most cells, while high expression of Arf1, Rab1, and ARL3A possibly suggest a considerable emphasis on flux through the ER and Golgi complex and flagellum biogenesis respectively [53-55]. Developmentally expressed small GTPases are restricted to the BSF. This cohort includes markers for the recycling endosome, Rab11 [15], the endosomes/Golgi, TbArf1 [53], and the flagellum, TbArl3A [55]. Identification of Rab11 is consistent with earlier work [15] and our data provide the first evidence for developmental regulation of TbArf1 and TbArl3A. The data also identify Rab1B, RabX3, Ras-like GTPases TbRX3 and TbRHP, and a putative GTPase with an Hsr1 domain (Tb927.4.2380) as developmentally regulated; with no experimental information for these proteins the significance of these changes is not clear [29,56]. Importantly, we see no strong developmental regulation for any of the putative GTPase-activating proteins (GAPs) and guanine nucleotide exchange factors (GEFs) included in the study. Some developmental regulation of TBC family Rab-GAPs is detectable by qRT-PCR, but at levels unlikely to be detected by the array (C. Gabernet-Castello and MCF, unpublished data).

In addition to a number of small GTPases, including Rab subfamily members, five predicted SNARE mRNAs exhibit increased expression in BSFs (Table 1). While no direct experimental information is available for these molecules, Tb09.211.3920, the most abundant Qa SNARE at the mRNA level (Figure 3), is likely involved in post-Golgi transport on account of similarity to *trans*-Golgi network (TGN) SNAREs SYP42 from *Arabidopsis thaliana *and syntaxin16/Tlg2p from *S. cerevisiae *[57,58], while the *L. major *orthologue, LmjF35.2720, localises close to the Golgi complex [59]. Tb10.70.7410 and Tb09.160.2420 are similar to the *A. thaliana *early endosomal SNARE VAMP727 and the ER-located SYP72, respectively [58], while Tb927.7.6440 and Tb927.3.3720 do not display significant similarity to any specific SNARE. By contrast, Tb10.61.0870, is the only upregulated SNARE message in the procyclic stage, with weak similarity to *Aspergillus *Bos1, suggesting a putative role in anterior transport from the ER; Tb10.61.0870 is also the most highly expressed Qb SNARE (Figure 3). These data are suggestive of specific upregulation of mRNAs encoding endocytic and exocytic SNAREs in BSF, with evidence for limited PCF regulation of the secretory pathway. On average, Qc and R SNAREs are more highly expressed than Qa and Qb SNAREs (Figure 3). The *T. brucei *N-ethylmaleimide sensitive factor (NSF, Tb927.1.1560), an ATPase responsible for regulation of SNARE complex disassembly [60], and a hypothetical protein similar to γ-soluble NSF attachment protein (γ-SNAP, Tb10.70.4620) are upregulated in BSF, which may indicate increased overall SNARE activity, but a further putative SNAP (Tb927.1.3110) is upregulated in PCF. Interestingly, both Tb927.1.1560 (NSF) and Tb927.1.3110 (SNAP) have severe RNAi knockdown growth phenotypes [61]. Expression of Vps16, a component of the HOPS tethering complex functioning at the late endosome, is increased in the BSF (Table 1). Overall, multiple factors likely to be controlling endocytic pathways, encompassing SNAREs, Rabs and possibly also the HOPS complex, appear more highly expressed in the BSF, consistent with earlier data but also suggesting more extensive remodelling than previously suspected.

#### Coat proteins

The vast majority of vesicle coat factor mRNAs are expressed at equivalent levels in both major life stages. Of those that are regulated, most notable is the clathrin light chain, Tb10.61.1910, upregulated in BSF and in agreement with increased endocytosis in this life stage [16]. Further, Tb11.01.6880, containing a predicted emp24 domain, suggesting a role as a cargo receptor in COPII-mediated transport [62], is upregulated in BSF, whereas a second emp24-related ORF, Tb927.4.4350, is upregulated in PCF. Should these proteins indeed represent emp24 isoforms, this expression profile indicates potential developmental-stage specific remodelling of COPII cargo selection providing differentiation in ER exit pathways.

A putative epsinR (Tb11.50.0006), is also significantly upregulated in BSF; epsinR is associated with clathrin coated vesicles and AP1 [63]. The AP1 subunit is significantly upregulated in BSF, but the μ-subunit of AP3, is upregulated in PCF (Table 1); both complexes have a role in endosome to TGN transport. Although no further adaptin components exhibit highly significant differential expression, a general trend of upregulation of AP1 component in BSFs and downregulation of AP3 and AP4 was evident on the microarray [see Additional file 3] which suggests developmental regulation. From previous analyses, the AP1 γ-subunit is constitutively expressed [64], but the β-chain is upregulated in BSF [16], suggesting possible subunit-specific regulation. To further confirm the significance of these data polyclonal antibodies were raised against the δ-subunit of AP3. Western blotting confirmed significant developmental regulation of AP3δ, with expression greatly augmented in the PCF [see Additional file 3]. These data are also consistent with evidence suggesting increased emphasis on lysosomal trafficking in the PCF [26,65].

#### Proteases

Several proteases are known components of the trypanosome surface and endocytic system or predicted to be, based on the presence of a signal peptide. The gp63 isoform TbMSP-B (Tb927.8.1610), is upregulated in PCF, while TbMSP-C (Tb10.70.5290) is upregulated in BSF (Table 1), confirming previous analyses [52]. TbMSP-A, previously identified as BSF-specific [52], is only slightly upregulated by microarray, but is validated strongly by qRT-PCR (results not shown). Further, metacaspase MCA3 (Tb927.6.930) showed significant upregulation in BSF, also confirming earlier data [66]. Microarray data indicate that MCA5 (Tb09.211.4760) is upregulated in PCF, but qRT-PCR data failed to validate this, suggesting more equivalent expression, also in agreement with earlier work [66]. Two subtilysin-like serine peptidases (Tb927.3.4230, Tb11.02.1280), a CAAX prenyl protease (Tb09.211.0680), and a thimet oligopeptidase A (Tb927.7.190) are upregulated in BSF. The thimet oligopeptidase Tb927.7.190 was recently suggested to play a role in dysregulated kinin metabolism observed in the plasma of trypanosome-infected hosts and to contribute to vascular lesions observed in African trypanosomiasis [67], consistent with augmented expression in BSF.

The most abundant protease class message is for Tb927.6.960, an L-cathepsin-like cysteine peptidase, and likely a lysosomal protein [68], which appears equivalently expressed in both life stages (Figure 3). Some of the developmentally regulated proteases are also abundant at the mRNA level (Figure 3), namely MSP-B (Tb927.8.1610), MCA5 (Tb09.211.4760), a subtilysin-like serine peptidase (Tb927.3.4230), and thimet oligopeptidase A (Tb927.7.190). Hence the major proteolytic activity likely resides within the cysteine protease class, as previously reported [69], but these new data suggest a significant level of developmental remodelling, as well as potential contributions from additional protease classes.

#### Chaperones and ER lumenal proteins

Several putative ER chaperones, likely to aid folding of secreted coat proteins, were also studied. Of these, Tb927.7.5790 is homologous to ERp44, a thioredoxin of the protein disulfide isomerase (PDI) family, which may have a role in controlling the redox state of the ER, and is upregulated in BSF [70]. Two additional PDI proteins are also upregulated in the bloodstream stage of *T. brucei *[71,72], suggesting remodelling of these factors between life stages.

In the PCF stage, two predicted mitochondrial chaperones, an Hsp70 (Tb927.6.3740) and an Hsp60 (Tb10.70.0280), are upregulated, consistent both with previous data [73,74] and increased mitochondrial activity [6]. Additionally, an Hsp70 (Tb11.01.3110) with high similarity to human Hsc70, an uncoating ATPase for clathrin coated vesicles, is upregulated in PCF [75-77]. Since clathrin-mediated endocytosis is upregulated in BSF but lysosomal targeting upregulated in PCF, these data may indicate a role in lysosomal targeting, but there are multiple Hsp70 ORFs in *T. brucei *and Hsp70 has been previously reported to be upregulated slightly in BSF [78]. Two novel ER lumenal proteins, Tb11.01.2640 and Tb11.01.8120, with a predicted signal peptide and C-terminal HDEL ER-retrieval motif are developmentally regulated, the former with augmented expression in BSF and the latter in PCF. Both are localised to the ER, and give a severe growth phenotype by RNAi knockdown, suggesting an important function within the ER (T. Sergeenko and MCF, unpublished data). Overall, the expression profile of the chaperones does not provide evidence for substantial differential remodelling of the ER folding environment, and may suggest a comparatively similar set of factors are required for synthesis of the cell surface in PCF and BSF, despite major differences in the structures of the superabundant surface antigens.

#### Lipid kinases

Two highly conserved lipid kinases with roles in intracellular trafficking are known in trypanosomes: Fab1 (Tb11.47.0002), a PI 5-kinase and TbVps34 (Tb927.8.6210), a PI 3-kinase involved in endocytosis and Golgi segregation [35,46]. Neither of these factors shows evidence for differential expression, but several other predicted phosphatidylinositol kinases are developmentally regulated (Table 1). A type III phosphatidylinositol 4-kinase, Tb927.3.4020, highly similar to *S. cerevisiae *Stt4p, is upregulated in PCF, consistent with a recent report of a role for a type III PI 4-kinase in protein trafficking, maintenance of Golgi and cell morphology and cytokinesis in PCFs [79]. A predicted phosphatidylinositol 3-kinase, Tb10.6k15.2060, is also upregulated in PCF and most likely represents an atypical protein kinase of the TOR-related family [35]. Significantly, human FRAP1, a member of the TOR-related kinase family, is required for clathrin-mediated endocytosis [18]. Tb11.01.6980, an atypical PI 3-kinase with a PI(3)P-binding FYVE domain and a putative N-terminal *trans*-membrane helix is highly upregulated in BSF; on account of the FYVE domain this protein is expected to localise to the endosomal system [35]. Further, Tb10.70.2440, a predicted PI4P 5-kinase, is also upregulated in BSF, while two serine threonine kinases with pleckstrin homology (PH) domains, capable of recognising PI4,5P lipids, are upregulated in BSF. Taken together these data suggest significant remodelling of the PI-based signalling system between life stages, together with evidence for increased expression of several putative endosomal functioning PI-kinases in the BSF.

#### Protein kinases

A genome-wide analysis of the roles of kinases in clathrin- and caveolin-mediated endocytosis was performed recently in *H. sapiens *[18]; significantly, caveolin-mediated endocytosis is restricted to the metazoa [46]. We looked for orthologues of the kinases that gave an endocytosis phenotype in the Pelkmans *et al *. study [18] and included these in the array to analyse their differential expression.

Two metabolic pathway kinases and three putative signalling kinases are upregulated in BSF. These include Tb10.70.5800, a glycosomal hexokinase, expected due to increased glycolytic activity in BSFs [14,80]; this kinase is similar to *H. sapiens *hexokinase II, required for clathrin-mediated endocytosis [18]. Pyruvate kinase (Tb10.61.2680) is also upregulated, in agreement with earlier work [81], and interestingly the related *H. sapiens *kinase, PKLR, inhibits caveolae-mediated endocytosis [18]. The three signalling kinases upregulated in BSF all show similarities to endocytosis kinases [18]. Tb927.6.1780, a putative MAP-kinase similar to human MAPK8, is activated in response to temperature stress [34]. A MAP-kinase kinase kinase, Tb10.61.2490, has similarity to human MAP3K8, and finally a calcium-dependent protein kinase, Tb927.4.3770, is highly similar to CAMK4, which participates in a calcium/calmodulin-dependent protein kinase cascade [82]. These data clearly suggest a role for kinase activity in BSF trafficking.

Eight primarily metabolic pathway kinases are upregulated in PCF. One is a nucleoside diphosphate kinase (Tb11.01.7800) which is involved in the equilibration of cellular pools of nucleotide triphosphates, and which binds telomeres and acts as a Rho-GAP in other systems [83-85]. This protein localises predominantly to the nucleus in *T. brucei *[86] and shows similarity to human NME6, found to inhibit clathrin-mediated endocytosis [18]. A ribokinase (Tb11.03.0090) upregulated in PCF is highly similar to human RBSK, required for clathrin-mediated endocytosis [18]. A putative NEK kinase (Tb927.3.3190) is related to human NEK2, 6, and 7, all of which are required for clathrin-mediated endocytosis [18], and a putative UTP-glucose-1-phosphate uridylyltransferase 2 (Tb10.389.0330) is related to human UGP2, required for clathrin-mediated endocytosis [18]. A glycosomal glycerol kinase (Tb09.211.3540) related to human CARKL, which is required for caveolin/lipid raft-mediated endocytosis [18], was upregulated in PCF, although equivalent levels of specific activity for this enzyme have been reported for both life stages [80,87]. An arginine kinase (Tb09.160.4560) involved in the management of cellular ATP energy reserves [88] is similar to human CKB, required for clathrin-mediated endocytosis [18]. A putative dephospho-CoA kinase (Tb927.6.710) which catalyzes the final step in CoA biosynthesis, is related to human DPCK, which is required for caveolin/lipid raft-mediated endocytosis [18]. A phosphoglycerate kinase (Tb927.1.720), similar to human PGK1, which is involved in both clathrin-mediated and caveolin/lipid raft-mediated endocytosis [18] was up in PCF, consistent with previous analyses for *T. brucei *PGKB [89], although the oligonucleotide used does not allow us to differentiate PGK isoforms. A putative Ser/Thr and Tyr dual specificity phosphatase (Tb10.406.0240) possibly involved in MAP kinase signalling, is also upregulated in PCF. Taken together these data suggest multiple kinases are involved in control of trafficking in both life stages, with more signalling kinases upregulated in BSF, and these data provide a number of candidates for further analysis.

#### Ubiquitylation

Covalent attachment of ubiquitin to surface molecules is an important mechanism for internalization and lysosomal targeting [90,91], and is present in trypanosomes [92,93]. Five ORFs likely to participate in the trypanosome ubiquitylation system are upregulated in BSF; Tb09.211.3610, an ubiquitin-activating E1 enzyme, Tb927.4.2710, an ubiquitin-conjugating E2 enzyme, Tb927.6.2370, an ubiqutin ligase E3, Tb11.46.0014, an ubiquitin carboxyl-terminal hydrolase similar to *S. cerevisiae *Doa4p protein, and Tb10.70.1130, which contains two putative ubiquitin interaction motifs, but otherwise has no predicted function. Upregulation of a near complete pathway for protein ubiquitylation and deubiquitylation in BSFs is suggestive of stage-specific ubiquitylation, but clearly direct evidence is required [94]. RNAi of the E1 Tb09.211.3610 shows a strong growth defect but does not affect the stability of ISG65, a surface protein subject to ubiquitylation [93]. A protein containing a VHS domain (Tb11.01.2540) which is normally found in the Vps27 component of ESCRT 0, and a putative Vps46 component of the ESCRT III-associated complex (Tb11.02.0580) are upregulated in procyclics, further supporting differential regulation of the ubiquitin sorting system.

#### Hypothetical open reading frames

A considerable fraction of the analysed ORFs are annotated as hypothetical, and were included on account of sequence features shared with characterized trafficking factors. Several of these exhibit developmental regulation. For example, the C2 domain is a Ca^2+^-dependent membrane-targeting module [95-97]; two ORFs with predicted C2 domains, Tb09.160.3240 and Tb927.7.3550, are upregulated in procyclics, and one, Tb927.4.2080, is upregulated in BSF. Interestingly, Tb927.7.3550 localises between the inner face of the plasma membrane and the sub-pellicular corset of microtubules in *T. brucei*, and includes a highly charged region characteristic of a tubulin-binding domain [98]. RNAi evidence indicates a clear role for Tb927.7.3550 in cytoskeletal function [98].

Other hypothetical genes that show significant differential regulation at the mRNA level are, in PCF: Tb927.7.2640, which contains part of a Sec34 domain (although it is not a true COG3/Sec34 orthologue [41]), and Tb11.47.0022, which contains a signal peptide and a *trans*-membrane helix; both ORFs are highly conserved between trypanosomatids but have no identifiable orthologues in other organisms. In BSF: Tb11.01.4460, which contains a cysteine-rich repeat region found in the low-density lipoprotein (LDL) receptor, and Tb927.5.4520, an extended coiled-coil protein are upregulated. The flagellum adhesion glycoprotein fla1 was upregulated in PCF, as has been reported previously [99], but the closely related fla2 [100] was upregulated in BSF, indicating that the two gene products may have similar functions but distinct expression patterns.

#### Surface antigens

Messages for BSF-specific ESAG6/7 and ISG65/75 surface antigens are highly abundant (Figure 3). By contrast, CRAM, a possible lipoprotein receptor abundant in PCF, and BARP, a putative glycolipid-lipid raft associated protein are not so highly expressed (Figure 3). Interestingly, some of the oligonucleotides for BARP showed upregulation in PCF and some in BSF (Table 1); BSF-specific and epimastigote-specific BARP expression have been reported previously [12,44]. The differential expression levels for the ISG75 mRNAs (Figure 3) represent mostly the degeneracy of each oligonucleotide [see Additional file 2], but the mRNAs for the ISG65 genes on chromosome 5 (Tb927.5.1390-1410-1430) are clearly more abundant than those for the ISG65 genes on chromosome 2 (Tb927.2.3280-3290-3300-3310). We also included oligonucleotides for several ISG-like proteins, four of which, Tb927.5.310, Tb927.5.420, Tb927.5.430, and Tb927.5.630, are significantly upregulated in BSF, suggesting additional and so far uncharacterised developmental modification of the parasite surface.

### Influence of stress and growth conditions on the transcriptome

The detection of a significant cohort of developmentally regulated trypanosome mRNAs by microarray, and their validation by qRT-PCR, provided confidence that our procedure could be exploited for investigation of transcriptome changes associated with differing conditions. In several model organisms, the interactions between gene products has been probed by analysis of coordinated changes to the transcriptome in response to challenges to the cell. Such studies have included several changes to growth conditions, insult with specific chemicals to invoke the unfolded protein response (UPR), and targeted manipulations of gene expression [101-104]. Here we used all three approaches to probe the *T. brucei *transcriptome in order to determine the level of flexibility, and to assess if such data could facilitate construction of interaction and pathway maps based on coordinate expression profiles.

#### Iron starvation

**Figure 4:**
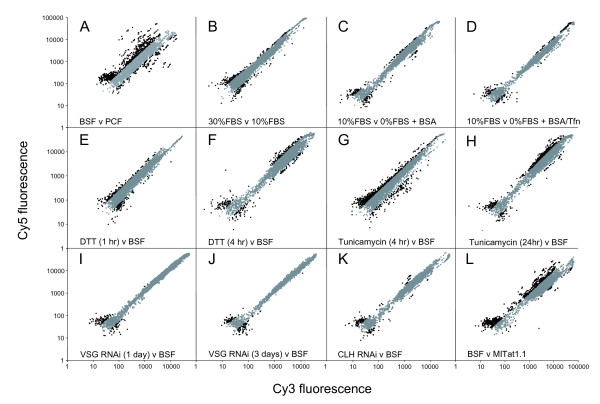
**Scatter plots of transcriptome data from trypanosomes subjected to altered culturing conditions and other challenges**. In each case, Cy5 fluorescence is plotted on the Y-axis and Cy3 fluorescence on the X-axis. Spots with a Cy5/Cy3 or Cy3/Cy5 ratio above two, i.e. significantly differentially expressed are highlighted in black, spots with a ratio below two are shown in grey. Panel A: BSF (Cy5) vs PCF (Cy3); data are identical to Figure 1 and reproduced for comparison. Panel B: BSF in 30% FBS (Cy5) vs BSF (Cy3). Panel C: BSF (Cy5) vs BSF in 0% FBS, 5 mg/ml BSA (Cy3). Panel D: BSF (Cy5) vs BSF in 0% FBS, 5 mg/ml, BSA 0.3 mg/ml Tfn (Cy3). Panel E: BSF in 1 mM DTT for 1 hr (Cy5) vs BSF (Cy3). Panel F: BSF in 1 mM DTT for 4 hr (Cy5) vs BSF (Cy3). Panel G: BSF in 5 μg/ml tunicamycin for 4 hr (Cy5) vs BSF (Cy3). Panel H: BSF in 5 μg/ml tunicamycin for 24 hr (Cy5) vs BSF (Cy3). Panel I: VSG RNAi in BSF, induced for 24 hr (Cy5) vs uninduced (Cy3). Panel J: VSG RNAi in BSF, induced for 72 hr (Cy5) vs uninduced (Cy3). Panel K: CLH RNAi in BSF, induced for 24 hr (Cy5) vs uninduced (Cy3). Panel L: BSF (Cy5) vs MITat1.1 grown *in vivo *in rats (Cy3). The scatter plots for most experiments (panels B-K) indicate few transcripts that fall outside of the region considered as constitutive, in marked contrast to the scattergrams obtained for developmental changes (panel A) and in the comparison between *in vitro *versus *in vivo *BSF cultures (panel L).

Bloodstream trypanosomes are sensitive to iron starvation, and upregulate ESAG6/7 transferrin receptor (TfR) mRNA and protein levels when placed in low transferrin or iron-depleted media [45,105] and adjust TfR expression to compensate for reduced endocytosis [65]. Both observations suggest a specific transcriptional-level response resulting from an iron-sensing mechanism. The transcriptomes of BSFs cultured in different proportions of fetal bovine serum (FBS), in the absence of FBS or in the absence of FBS but supplemented with bovine *holo*-transferrin were analysed. An increase in ESAG6/7 mRNA was detected under FBS depletion by the array (Table 2), which was validated by qRT-PCR in agreement with previous analyses [45,105]. mRNAs for procyclin, the gp63 isoform TbMSP-C (Tb10.70.5290) and the major cysteine peptidase (Tb927.6.960) were upregulated in serum-depleted cells (Table 2). In cells grown in 30% FBS, procyclin mRNA, as well as an ISG-like protein (Tb927.5.410) and a putative calcium-dependent protein kinase (Tb927.4.3770) were downregulated (Table 2), which contrasts with a moderate upregulation of procyclin in serum free conditions. These alterations were confirmed by qRT-PCR, and may suggest a suppression of procyclin expression by serum factors. However, overall there were no additional transcriptome changes when cells were grown in 30% FBS or in 0% FBS with or without transferrin. Scatter plots of the entire array data set indicate few transcripts that fall outside of the region considered as equivalent, in marked contrast to the scattergram obtained for developmental changes (compare Figure 4B–D with Figure 4A or Figure 1). These data suggest a very limited and specific ability of the BSF to respond to alterations in levels of nutrients, which may be restricted primarily to ESAG6/7.

**Table 2:** Expression changes accompanying environmental challenge in *T. brucei*.

			Fold up (down) vs control	
			
Experiment	Gene	Accession	array	qRT-PCR
**SMB grown with 0%FBS 5 mg/ml BSA vs with 10% FBS**	Gp63 (TbMSP-C)	Tb10.70.5290	**2.9**	2.2
	procyclin	Tb10.6k15.0030	**2.4**	2.4
	procyclin	Tb927.6.450	**2.0**	2.3
	cysteine peptidase	Tb927.6.960	1.9	3.6
	ESAG6	H25N7.15	**1.8**	3.9
	ESAG7	N19B2.155	**1.6**	4.7
	14-3-3-like	Tb11.02.4700	**(1.9)**	

**SMB grown with 0%FBS, 5 mg/ml BSA, 0.3 mg/mg Tfn vs with 10% FBS**	Gp63 (TbMSP-C)	Tb10.70.5290	**3.1**	1.5
	procyclin	Tb10.6k15.0030	**2.2**	1.9
	cysteine peptidase	Tb927.6.960	2.0	4.6
	ESAG6	H25N7.15	2.0	4.1
	ESAG7	N19B2.155	1.4	3.5

**SMB grown with 30%FBS vs with 10% FBS**	ISG65-like	Tb927.5.410	**(2.9)**	(4.1)
	CAMK4 kinase	Tb927.4.3770	**(2.2)**	
	procyclin	Tb10.6k15.0030	**(1.9)**	(3.6)

**SMB grown for 1 hr with 1 mM DTT vs without**	GTP1/OBG/Hsr1	Tb927.2.5060	**2.4**	
	putative ABC transporter	Tb10.329.0040	**2.2**	
	ISG65-like	Tb927.5.410	**(2.9)**	(1.9)
	ESAG7	N19B2.155	**(2.2)**	(2.0)
	MAP kinase	Tb10.70.2070	(2.1)	
	Rab3GAP	Tb10.6k15.1390	**(2.1)**	
	dual-specificity protein phosphatase	Tb927.5.3620	**(1.9)**	

**SMB grown for 4 hr with 1 mM DTT vs without**	TbGRP	Tb10.6k15.1520	**3.6**	
	hypothetical ER chaperone	Tb11.01.8120	**3.0**	3.3
	Ubiquitin conjugating	Tb927.7.6960	**2.8**	
	Vta1-like	Tb927.7.7140	**2.7**	
	syntaxin	Tb10.61.1980	**2.7**	
	Hsp60 chaperonin	Tb10.70.0280	**(3.0)**	
	GMP-PDE delta	Tb927.2.4580	**(2.8)**	
	Rab11a	Tb927.8.4330	**(2.8)**	(1.8)
	Ser peptidase (Bem46-like)	Tb09.211.2310	**(2.4)**	
	Sec24	Tb927.3.5420	**(2.4)**	

**SMB grown for 4 hr with 5 ug/ml Tunicamycin vs without**	procyclin	Tb10.6k15.0030	2.4	2.1
	ESAG6	H25N7.15	1.9	2.3
	procyclin	Tb927.6.450	1.7	2.3
	AP2-kinase1	Tb09.160.4770	(2.0)	

**SMB grown for 24 hr with 5 ug/ml Tunicamycin vs without**	procyclin	Tb10.6k15.0030	4.2	7.8
	procyclin	Tb927.6.450	2.7	8.8
	ESAG7	N19B2.155	2.6	4.3
	ESAG6	H25N7.15	2.2	2.8
	RabGAP	Tb10.6k15.1790	2.1	
	GOLD domain	Tb927.7.3600	2.1	
	PDI/ERp72	Tb10.6k15.2290	(2.3)	

**VSG RNAi induced vs uninduced Day1**	RabGEF/Vps9	Tb927.3.2430	**2.1**	

**VSG RNAi induced vs uninduced Day3**	nothing			

**CLH RNAi induced vs uninduced 24 hr**	Gp63 (TbMSP-C)	Tb10.70.5290	**2.7**	3.6
	ISG65-like	Tb927.5.410	**2.8**	3.8
	CLH	Tb10.70.0830	**(2.5)**	
	GMP-PDE delta	Tb927.2.4580	**(2.3)**	
	CBL-interacting protein kinase 9	Tb927.8.870	**(2.0)**	

**Rab5AWT overexpressing PCF line vs PCF WT (both with 20% FBS)**	alpha tubulin	Tb927.1.2400	**2.9**	1.7
	histone H3	Tb927.1.2430	**2.7**	1.5
	Rab5a	Tb10.389.0550	**2.6**	9.5
	RabX3	Tb11.01.6670	**2.5**	3.5
	40S ribosomal protein S25	Tb927.3.1370	**2.2**	1.2
	Amphiphysin	Tb11.01.6740	**2.2**	
	Hsp70	Tb927.6.3740	**2.0**	
	cysteine peptidase	Tb927.6.960	**2.0**	
	COPI epsilon	Tb11.01.6530	**2.0**	2.6
	adenylate kinase 3	Tb10.70.5150	**2.0**	3.1
	CBL-interacting protein kinase 9	Tb927.8.870	**(2.3)**	1.6
	Ubiquitin conjugating	Tb11.02.0815	**(2.1)**	1.6
	ARPC5	Tb10.406.0320	**(2.0)**	

**Rab5AQL overexpressing PCF line vs PCF WT**	histone H3	Tb927.1.2430	**2.3**	1.6
	40S ribosomal protein S25	Tb927.3.1370	**2.1**	1.1
	Rab5a	Tb10.389.0550	**2.0**	9.8
	adenylate kinase 3	Tb10.70.5150	**1.6**	3.8
	alpha tubulin	Tb927.1.2400	**1.6**	2.1
	COPI epsilon	Tb11.01.6530	1.4	3.0
	RabX3	Tb11.01.6670	1.3	3.4
	mitochondrial intermediate peptidase	Tb10.6k15.0490	**(2.4)**	
	mitochondrial zinc metallopeptidase	Tb927.4.3300	**(2.0)**	

**Rab5BQL overexpressing PCF line vs PCF WT**	Rab5b	Tb11.02.2160	**2.5**	16.7
	adenylate kinase 3	Tb10.70.5150	**1.6**	3.5
	COPI epsilon	Tb11.01.6530	**1.5**	3.4
	RabX3	Tb11.01.6670	**1.5**	2.8

The most significantly altered mRNAs detected by microarray for differing serum conditions, dithiothreitol and tunicamycin treatment, RNAi of VSG or CLH, and Rab5 overexpressing PCF lines are shown. The ratios obtained by qRT-PCR for selected transcripts are also shown. Ratios are given on a linear (not log) scale, and those indicating a reduction compared to the control state are in parentheses. Only ORFs that display at least a two-fold change, by either qRT-PCR or array, are included; except the 4 hour DTT treatment, where only the top ten hits are shown. Ratios which correspond to a value >1 for the B-statistic in limma analysis are shown in bold.

#### Unfolded protein response (UPR)

Transcriptome responsiveness was further tested by attempts to invoke the UPR, a classic ER-based pathway stimulated by the presence of increased levels of unfolded polypeptides [106]. The UPR is mediated via transcriptional responses in both yeast and metazoan cells [104,107], and can be reliably activated by addition of tunicamycin or dithiothreitol (DTT). Using tunicamycin and DTT at concentrations that invoke a UPR in mammalian cells, *Arabidopsis*, yeast and other systems [108-112], we found that, in *T. brucei*, tunicamycin resulted in growth arrest over a period of up to 24 hours, while DTT exposure led to rapid cell death [see Additional file 4]. This concentration of tunicamycin also efficiently inhibits trypanosome N-glycosylation [113]. Therefore, we analysed the transcriptome at 5 μg/ml and 1 mM for tunicamycin and DTT, respectively, under conditions where cells remained viable, as assessed by motility.

Essentially no significant change to the transcriptome was observed by either treatment. Very few ORFs showed significant differential regulation under DTT or tunicamycin treatment, and scatterplots indicate few transcripts falling outside of the equivalence region, in marked contrast to developmental changes (Table 2, Figure 4E–H). Only one hypothetical chaperone was upregulated after 4 hr in 1 mM DTT, while Rab11, ESAG7, and an ISG-like protein (Tb927.5.410) were downregulated. By contrast, a PDI class ORF, Tb10.6k15.2290, was downregulated after 24 hr of tunicamycin treatment, while ESAG6/7 as well as procyclin were upregulated in cells treated with tunicamycin (Table 2). Further, total cell lysates were examined for increased expression of the major ER chaperone, BiP, by Western blotting and qRT-PCR. No alteration in expression of this marker, which is rapidly and strongly induced by the UPR in higher eukaryotes, was observed following DTT or tunicamycin exposure [see Additional file 4]. These data indicate an essential lack of transcriptome responsiveness to altered conditions by the trypanosome, and specifically the complete absence of a classical UPR. Significantly, this likely contributes to the extreme sensitivity of trypanosomes to DTT.

#### Knockdown of clathrin and VSG

As an additional strategy for investigating transcriptome responsiveness, the mRNAs of two highly important proteins involved in endocytosis and surface architecture, specifically the clathrin heavy chain (CLH) and VSG, were suppressed with RNAi. Both proteins are essential and CLH RNAi results in rapid cell death. For CLH, knockdown leads to a complete block to endocytosis [114], while VSG RNAi results in arrest of cell cycle progression [115]. We hypothesized that if trypanosomes were able to sense alterations in trafficking and respond to these changes, then depletion of these two ORFs by RNAi would be expected to elicit a response.

We analysed the transcriptome of cells 24 hours after induction of the CLH RNAi, as the CLH protein levels are significantly reduced at that stage [see Additional file 5], growth is arrested, and the big eye morphology is clearly visible (data not shown). Despite a clear phenotype, we observed very limited transcriptome changes (Figure 4K, Table 2). Only TbMSP-C and an ISG-like ORF (Tb927.5.410) were significantly upregulated, while only a putative Rab effector, GMP-PDEδ (Tb927.2.4580) and a putative CBL-interacting protein kinase (Tb927.8.870) were significantly downregulated (Table 2). For the VSG RNAi, we analysed cells 24 and 72 hours after induction to examine both early and late effects of VSG depletion. No major transcriptome changes were observed (Figure 4I–J, Table 2) despite growth arrest [see Additional file 5]. Indeed, only one ORF, a putative RabGEF (Tb927.3.2430) was significantly upregulated (Table 2). The absence of a coordinated response within the transcriptome as a result of these severe perturbations of cellular homeostasis suggests that trypanosomes lack mechanisms for response at the mRNA level to altered circumstances.

#### The transcriptome is unaltered in cells with augmented endocytic activity

**Figure 5:**
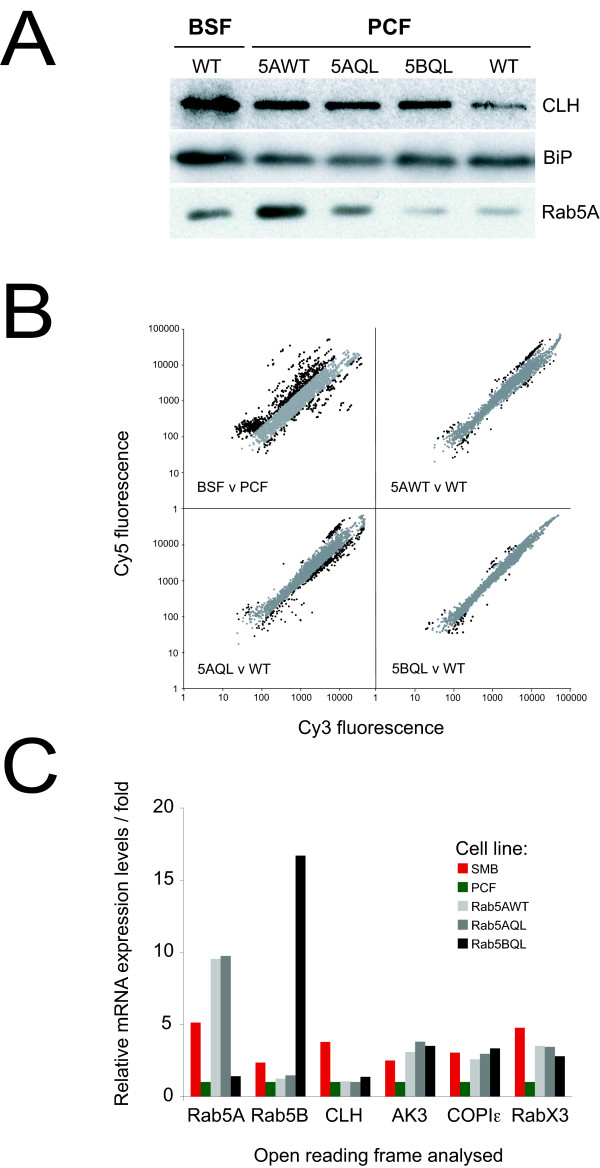
**Limited differential expression in Rab5-overexpressing PCF cells**. Panel A: Western blot analysis for the clathrin heavy chain (CLH) and Rab5A, for wildtype BSFs and PCFs, PCFs overexpressing Rab5A (5AWT), Rab5A^QL ^(GTP-locked mutant) and Rab5B^QL ^(GTP-locked mutant) cell lines. BiP was used as a loading control. Panel B: Scatter plots of raw data for representative microarrays for BSF (Cy5) vs PCF (Cy3) experiment (plot as in Figure 1 for comparison), and Rab5-overexpessing lines (Cy5) vs PCF (Cy3) as indicated. Cy5 fluorescence is plotted on the Y-axis and Cy3 fluorescence on the X-axis. Spots with a Cy5/Cy3 or Cy3/Cy5 ratio above two are highlighted in black, spots with a ratio below two are shown in grey. Panel C: Relative expression levels of Rab5A, Rab5B, CLH, RabX3, COPIε, and adenylate kinase 3 (AK3) in wildtype SMBs and PCFs, and in the Rab5-overexpressor PCF lines, as assessed by qRT-PCR.

As a final test of transcriptome responsiveness we analysed the effects of over-expression of the early endosome small GTPases Rab5A and Rab5B in PCF, which results in significant augmentation of fluid phase and receptor-mediated endocytic activity [116]. The expression level of CLH was increased, providing a mechanistic explanation for augmented endocytic activity (Figure 5A). These data extend earlier work showing loss of CLH protein following Rab5A or 5B knockdown in BSF [117], and demonstrating coordinated expression of clathrin and Rab5.

Very limited differential expression was seen between Rab5 over-expressing and control cells (Figure 5B, Table 2). Analysis by array, qRT-PCR and Western blot confirmed that the cell lines were overexpressing Rab5A or Rab5B but only detected significant upregulation for RabX3, adenylate kinase 3 (Tb10.70.5150), and COPIε mRNAs in the Rab5A^WT^, Rab5A^QL ^and Rab5B^QL ^overexpressor lines (Table 2, Figure 5C), none of which are expected as specific changes related to early endosome activity. Further, several mRNAs implicated as altered by array analysis failed to be validated by qRT-PCR, suggesting small or insignificant changes only (Table 2). Importantly, we were unable to detect a significant increase in CLH mRNA in any of the cell lines by qRT-PCR, despite significant protein level changes (Figure 5). These data suggest, firstly, that alteration of CLH levels in these cells proceeds via exclusively post-transcriptional mechanisms and, secondly, that despite clear perturbation to endocytic activity, this is not reflected in an obvious or coherent alteration to the underlying transcriptome.

#### Significant transcriptome alterations between *in vivo *and *in vitro *cultured trypanosomes

The vast majority of molecular studies on *T. brucei *are carried out using *in vitro *cultures. Recently, whole-genome microarray analysis indicated significant differences between *in vivo *and *in vitro *cultures of the malaria parasite *Plasmodium falciparum *[118]. Therefore, as an additional investigation into the influence of culturing conditions on intracellular transport we compared mRNA from bloodstream form MITat1.1 trypanosomes extracted from infected rats and from SMB cells cultured under standard conditions. A significant number of differentially abundant mRNAs are evident from the scattergram of these arrays (Figure 4L), and limma analysis indicated that 35 ORFs are significantly upregulated in the *in vitro *culture, while three ORFs are significantly downregulated (Table 3). These include ORFs for an RNA helicase, procyclin, CAP5.5, several small GTPases and their effectors and several kinases (Table 3). This expression pattern did not reveal an obvious functional pattern, in the sense that ORFs from many biological systems appeared affected. To eliminate the possibility that these alterations arise from cold-shock during isolation of MITat1.1 trypomastigotes, we also extracted RNA from SMB cultures incubated at 4°C for 1 hr. We observed very few changes between normal and cold-shocked SMB cultures (Table 3), with only CAP5.5 and a Sec34-like ORF significantly upregulated in the cold treatment, both of which are procyclic markers. Given the rapidity of isolation of the mRNA from MITat1.1, which is achieved in less than one hour, and their incubation at 4°C during the procedure, which is expected to reduce metabolic activity, we consider these findings to be unlikely ascribable to factors associated with preparation of the samples as the differences in several mRNA levels are too great to have accumulated in such a short period. Therefore, these data highlight significant differences between *in vivo *and *in vitro *cultured trypanosomes; this issue would be best pursued by whole genome microarray analysis which is beyond the scope of the present study.

**Table 3:** Expression changes associated with *in vivo *versus *in vitro *culture of *T. brucei*.

**MiTat vs SMB**	Gene	Accession	Fold difference
up in SMB	DHH1	Tb10.70.3290	**6.2**
	procyclin	Tb927.6.450	**5.0**
	PRA1	Tb10.389.1550	**4.1**
	procyclin	Tb10.6k15.0030	**3.8**
	CSNK1D (casein kinase)	Tb927.5.800	**3.6**
	CAP5.5	Tb927.4.3950	**3.5**
	Rab1a	Tb927.8.890	**3.5**
	SNF1-like kinase	Tb10.70.1760	**3.5**
	cofilin (ADF)	Tb927.3.5180	**3.1**
	NEK2/6/7 kinase	Tb927.3.3190	**3.0**
	PI3K (FYVE)	Tb11.01.6980	**2.8**
	Trs20/sedlin	Tb927.8.5900	**2.7**
	Ser peptidase (Bem46-like)	Tb09.211.2310	**2.7**
	Ubiquitin conjugating	Tb11.02.0815	**2.6**
	AP4 sigma	Tb10.61.2530	**2.6**
	ARPC5	Tb10.406.0320	**2.6**
	FYVE Zn finger containing protein	Tb927.4.3380	2.5
	ARPC4	Tb927.2.2900	**2.5**
	GSK3B kinase	Tb927.7.2420	**2.5**
	IFT88	Tb11.55.0006	2.3
	RabGAP	Tb10.6k15.1770	**2.3**
	nuclear transport factor 2	Tb10.70.5500	**2.3**
	ISG65-like	Tb927.5.410	**2.3**
	two UIM domains	Tb10.70.1130	**2.2**
	t-SNARE	Tb11.03.0965	**2.2**
	Rab7	Tb09.211.2330	**2.2**
	CBL-interacting protein kinase 9	Tb927.8.870	2.2
	VHS domain containing protein	Tb11.01.2540	2.2
	GTP1/OBG/Hsr1	Tb927.2.4240	**2.1**
	Hsr1/GTP-binding	Tb927.7.2630	2.1
	MAPK8 kinase	Tb10.70.2070	**2.1**
	PABP2	Tb09.211.2150	**2.1**
	calreticulin (lectin)	Tb927.4.5010	**2.0**
	RanGEF	Tb927.8.7290	**2.0**
	ARL3A	Tb927.3.3450	**2.0**

up in MiTat1.1	p67	Tb927.5.1810	**2.0**
	polyUb	Tb11.01.1680	**2.0**
	RanGEF	Tb10.6k15.3150	**2.9**

**SMB 4°C vs SMB**	Gene	Accession	Fold difference

up in 4°C	CAP5.5	Tb927.4.3950	**2.3**
	Sec34-like	Tb927.7.2640	**2.0**

Significantly differentially expressed transcripts for *in vivo *MITat1.1 bloodstream culture vs *in vitro *SMB culture, and for SMB treated at 4°C for one hour. Ratios are given on a linear scale. Only ORFs which show at least a two-fold change are shown. Ratios which correspond to a value >1 for the B-statistic in limma analysis are shown in bold.

## Discussion

Coordinate control of gene expression is critical for life cycle progression in trypanosomes, and extensive remodeling of the parasite cell is well documented. Here we used the intracellular trafficking system as a model for transcriptome changes. We selected this process on account of well documented alterations to endocytosis accompanying developmental progression, a good level of characterization in trypanosomes, together with, by analogy to higher eukaryotes, the expectation that many gene products will collaborate in regulation. Endomembrane transport involves ~10% of the protein coding potential within the eukaryotic genome [119,120] and position and time-dependent targeting, assembly of macromolecular complexes and cytoskeletal interactions all participate in controlling a system demanding high fidelity in delivery of molecules to specific subcellular destinations. Further, in higher eukaryotes, transcriptional flexibility and coordination are associated with intracellular transport [17,121]. Due to the polycistronic mode of transcription in trypanosomes, changes in relative levels of mRNAs result primarily from alterations in the efficiency of nascent RNA processing or half-life [13]. Here we detected clear evidence for developmental alterations to the trypanosome transcriptome, but found very little flexibility within a given life stage.

Firstly, ~6% of transport-associated transcripts are developmentally regulated, a greater fraction than reported for genome-wide transcription [14]. These data indicate that developmental remodeling of endomembrane transport is underpinned by alterations to mRNA abundance, as expected. We also observed prominent differential expression of transport-associated mRNAs between trypanosomes cultured *in vitro *and those isolated from a mammalian host. A greater proportion of mRNAs were upregulated in the bloodstream stage compared to the insect form, correlating with increased endocytic activity. Prominent upregulated factors include Rab GTPases and SNAREs; significantly, simple upregulation of Rab and/or SNARE proteins is sufficient to augment specific transport pathways [116,122-124]. While the cohort of upregulated mammalian stage mRNAs is consistent with increased endocytic activity, there is evidence that the Rab5/Rab11 VSG endocytosis and recycling system [25,122,125] is only part of the developmental change. For PCFs, the upregulated cohort is consistent with lysosomal degradative pathways playing a more prominent role [26]. Direct examination of several of the gene products implicated here is clearly required for a more detailed understanding of trypanosome differentiation.

Secondly, and in clear contrast, extremely limited changes to mRNA levels were encountered in response to a wide variety of altered states, including major changes to culturing conditions, insult with agents disrupting protein folding and RNAi of critical mRNAs encoding factors essential for endocytosis and cell surface maintenance. Despite polycistronic transcription, a *priori *there is no compelling reason to assume that turnover of mRNA is uncoupled from signaling systems and, moreover, the observation that iron-deficiency leads to increased expression of the ESAG6/7 transferrin receptor mRNAs and protein provides a potential example of signal-mediated changes to mRNA abundance  [45,65,105]. Analysis of a large gene cohort indicates that the transferrin receptor is a special case, and we were unable to find compelling evidence for alterations to levels of most mRNAs. The transferrin receptor genes are located in an RNA Pol I-transcribed subtelomeric array [126] and thus may be regulated differentially from most genes that are transcribed by RNA Pol II [127]. We also observed mRNA level alterations in a further Pol I-derived transcript, procyclin, in response to several challenges, but we note that procyclin expression is unusually sensitive to many factors (for example [128]). Regardless of these exceptions, our data indicate that the vast majority of trypanosome mRNAs are unmodulated, suggesting that, for most messages, transcription and turnover are unaffected by signaling pathways. This implies that the trypanosome has a very limited capacity to respond to altered circumstances *in vivo*, except *via *pre-programmed differentiation pathways.

The absence of transcriptome responsiveness to environmental cues has several consequences. Firstly, lack of a classical UPR indicates ER insensitivity to increased concentrations of non-native polypeptides, and implies that changes in biosynthetic output similarly will not be sensed. This is consistent with extreme sensitivity to concentrations of DTT that are well tolerated in higher eukaryotes. Whether the UPR is absent from additional excavates is unknown, but preliminary evidence suggests that *Giardia *also lacks the pathway [112]. Secondly, failure to modify the transcriptome following gene knockdown or over-expression, but where a major impact on transport is found, suggests that modulation of trafficking, and potentially other systems, within a given life stage is restricted to proteomic changes [117]. Thirdly, and most significantly, the data indicate an inability of the trypanosome endomembrane system to adapt to altering conditions and, coupled with the possibility that such unresponsiveness extends to additional cellular functions, has profound implications.

**Figure 6:**
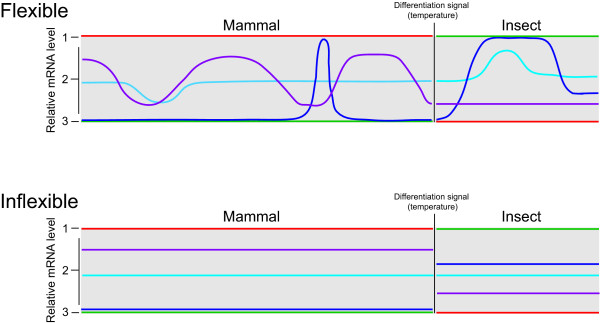
**Transcriptional flexibility and inflexibility in differentiation and responsiveness**. Upper panel: flexible system. Gene cohorts 1 and 3 are developmentally regulated, and either highly expressed or not expressed; examples of these types of gene products are the trypanosome surface antigens, VSG in the bloodstream form (red) and procyclin in the insect stage (green). The vast majority of genes fall into cohort 2, where, for example, either small or large changes to transcription could result from alterations to the environment (light and dark blue), or a more continually altering transcriptional profile is present that may seek to track changing conditions (purple). This behavior may propagate from one life stage to the next (light and dark blue) or be lost (purple) resulting in altered transcriptional flexibility for genes between life stages. Such a profile is found in higher eukaryotes, including humans and yeast, and probably also many protists, including *E. gracilis*. Lower panel: inflexible system. In this model gene cohorts 1 and 3 behave as before, but transcription of the genes in cohort 2 remains unchanged. The relative levels of mRNAs from the genes in this cohort may remain constant following differentiation (light blue) or be significantly altered (dark blue and purple). Such a profile is observed here for *T. brucei *and has been reported previously for *P. falciparum*, and is potentially a result of a parasitic life style where the host is responsible for provision of a homeostatic environment.

Limited transcriptional flexibility is clearly a restriction for the trypanosome, but is in agreement with a modest small GTPase signaling network [30], which may indicate a rather limited signal transduction capacity. As each life stage exists within a highly controlled environment the trypanosome's ability to modulate the trafficking system has likely been lost. The environment within the insect host is poorly understood, but the bloodstream pH range in *H. sapiens *is 7.35 – 7.45, blood glucose is at 4 – 8 mM and transferrin concentration is maintained at ~300 ± 50 μg/dl [129]; major alterations to these parameters are lethal, and hence there is little need for the parasite to retain mechanisms for responsiveness. By contrast, a new transcriptome would be triggered by appropriate signals when the trypanosome enters a new compartment or host. Hence the trypanosome life cycle may be viewed as a set of inflexible, sequential transcriptional profiles that serve to adapt the parasite to each new environment (Figure 6). The *in vitro *cultured BSF is a convenient model for the mammalian bloodstream trypomastigote but, during infection, trypanosomes also invade tissue spaces, the lymphatic system and, in later disease stages, the cerebral spinal fluid [1]. Each environment differs greatly from the bloodstream in composition and hence transcriptome inflexibility suggests that a differentiation event may be required for adaptation. Further, the major transcriptome differences between cultured and animal-isolated trypanosomes underscores the potential for currently uncharted, but dramatic, adaptations associated with mammalian infection.

A similar transcriptional rigidity is suggested for the intra-erythrocytic stages of the malaria parasite, *Plasmodium falciparum*, possibly also related to the fact that *Plasmodium *enjoys a homeostatic host environment [130-132]. Trypanosomatids (Excavata) and Plasmodia (Chromalveolata) are highly divergent and separated by many non-parasitic or free-living species, and thus transcriptome unresponsiveness most likely is the result of independent secondary loss accompanying adoption of the parasitic life style. Consistent with this view, environmental stress treatments do induce transcriptional responses in *Euglena gracilis *[133], a free-living protist closely related to kinetoplastids. Further, as *Plasmodium *utilises a more conventional promoter-mediated mechanism for transcription, this also rules out a trivial explanation that transcriptome inflexibility is simply the result of polycistronic transcription. Examination of transcriptome regulation in more free-living organisms related to both *Plasmodium *and trypanosomes would be highly informative.

Finally, these data indicate that knockdown of a specific mRNA does not alter the transcript levels of additional gene products coordinated functionally with the RNAi target. Therefore, in *T. brucei *RNAi is unlikely to suffer from off target effects as seen in higher eukaryotes [134-136], and hence is, in this regard, unusually clean. Secondary effects are likely mediated via proteome changes, and could explain the similar phenotypes observed for many knockdowns [61]. This is particularly prominent for cell cycle/cytokinesis defects that frequently emerge rather late following knockdown. Such effects may be the result of generic loss of the normal proteome, rather than absence of specific factors.

## Conclusion

The life cycle of *T. brucei *includes a dramatic differentiation event as the parasite progresses from the insect to the mammalian host. Differentiation encompasses considerable remodeling of the membrane trafficking system, which is also a vital component of the host-parasite interface. Using a subgenome microarray targeting the *T. brucei *trafficking systems together with qRT-PCR analysis, we find clear evidence for developmental alteration to ~6% of the trypanosome transport-associated transcriptome. This indicates that developmental remodeling of endomembrane transport is underpinned by alterations to mRNA abundance, and that such changes may be analysed by microarray. Most protein-coding mRNAs in trypanosomatids are produced by polycistronic transcription, with specific rates of decay as the major mechanism controlling steady state transcript levels. Multiple stimuli, including major changes to culturing conditions, insult with agents disrupting protein folding, and RNAi of critical mRNAs encoding factors essential for endocytosis and cell surface maintenance, elicited extremely limited changes to the transcriptome. Besides alterations in expression of the transferrin receptor upon iron stress, which is transcribed by Pol I, the vast majority of trypanosome chromosomal Pol II-derived mRNAs are unmodulated. These findings imply a very limited capacity of trypanosomes to respond to altered circumstances in vivo, except via pre-determined differentiation pathways (Figure 6). Hence mRNA levels are insensitive to external stimuli and most responses are mediated at the proteome level. These data suggest a new view of the trypanosome life cycle as a progression through sequential, inflexible transcriptional profiles that serve to adapt the parasite to each new host environment. As similar transcriptional rigidity is suggested for the malaria parasite, *Plasmodium falciparum*, but transcriptional flexibility is found in the closely related and free-living protist, *Euglena gracilis*, it is likely that loss of transcriptional flexibility arose through the parasitic lifestyle.

## Methods

### Open reading frame selection

We selected ORFs encoding components of the trypanosome transport system using the geneDB interface [137]. The genome was parsed with four criteria; (i) annotation keywords, including description information and wildcards, for protein classes involved in endocytosis and membrane trafficking (e.g. clathrin, adaptin, SNARE, Rab, ADP-ribosylation factor, coatomer, Sec, Vps), (ii) Pfam/Interpro domain annotation (e.g. ARF/SAR superfamily, C2, ENTH, PH, SH3, SNF7, VHS, BAR), (iii) "Biological Process" Gene Ontology (GO) annotation (e.g. endocytosis, vesicle mediated transport, GTPase activity) and (iv) sequence similarity by BLAST. The target gene selection was augmented by parsing the literature for candidate factors, i.e. yeast and mammalian proteins that have been functionally demonstrated to be involved in trafficking. We thus identified 673 ORFs for inclusion.

### Array construction

Oligonucleotides were designed with OligoArray 2.0 [138] specifying a 67 mer within one kilobase of the 3'-end of each target protein coding region. 694 oligonucleotides were designed, targeting 796 genes (several ORFs are multicopy and hence multiple transcripts are sampled by a single oligonucleotide in such cases). The array encompassed 56 oligonucleotides targeting 123 reference ORFs, whose expression pattern has been established previously. Oligonucleotides were manufactured by Illumina (Invitrogen) and included a 5' amino modification for attachment to Codelink activated slides (GE Healthcare). Arrays were printed using a BioRobotics Microgrid (Genomic Solutions) equipped with 16 NanoPins (Matrix Technology Corporation) in the Centre for Microarray Resources, Department of Pathology, University of Cambridge. Slides were prepared, washed and dried according to the manufacturer's instructions (GE Healthcare). Each oligonucleotide was spotted in quadruplicate, while α- and β-tubulin were spotted at the corners of the grid and reference oligonucleotides and additional spotting controls (empty, water, spotting buffer) were distributed randomly.

### Cells, in vitro and in vivo culture conditions

Cultures of *T. brucei brucei *Lister 427 single marker bloodstream form (SMB) and procyclic form (PCF) strain 427 were maintained in HMI-9 (Invitrogen) and SDM-79 (Sigma) media, respectively [139-141]. Rab5A and Rab5B over-expressing PCF lines have been described previously [116]. A VSG knockdown line was generated by expression of a double strand fragment of the 221 VSG ORF (PCR primers: TTTCTGCAGCGGTCACTATG and TGCTTGTTGTTGGCTTTCAG) cloned into *Eam*1105I-digested p2T7^TABlue ^[61]. Tetracycline-responsive SMB cells were transfected with *Not*I-digested p2T7^TABlue^-VSG by electroporation, selected and maintained with 2.5 μg/ml G418 and 2.5 μg/ml hygromycin. RNAi was induced by adding 1 μg/ml tetracycline. The clathrin RNAi BSF line was generated as described [114] except that the TbCLH gene fragment was subcloned into the p2T7^TA ^vector [114] and an Amaxa nucleofector was used for transfection [142]. Transformants were maintained under 2.5 μg/ml neomycin and 2.5 μg/ml hygromycin selection, and RNAi was induced by adding 1 μg/ml tetracycline. For treatment with dithiothreitol (DTT) and tunicamycin, SMB cells were grown in HMI-9 to 1 × 10^6 ^cells/ml, and the media supplemented with either DTT (Sigma) at 1 mM, or tunicamycin (Sigma) at 5 μg/ml. Samples for RNA extraction were taken after 1 hr and 4 hr for DTT addition and 4 hr and 24 hr for tunicamycin. To assess the influence of serum on gene expression, cells were grown in standard media until a concentration of 1 × 10^6 ^was reached and harvested by centrifugation at 800 g for 10 minutes. The cell pellet was re-suspended in media lacking fetal bovine serum (FBS), and supplemented with 5 mg/ml BSA with or without 0.3 mg/ml bovine *holo*-transferrin (Sigma), to simulate physiological concentrations of albumin and transferrin in media supplemented with 10% FBS [129]. For 30% FBS treatment cells were grown in HMI-9 media supplemented with 30% FBS.

For *in vivo *grown cells, the blood from rats infected with monomorphic bloodstream forms (MITat1.1 at 1 × 10^9 ^cells/ml parasitemia) was taken by aortic puncture into heparinized syringe. The buffy coat fraction was prepared by centrifugation of infected blood (600 g, 10 min at 4°C). All subsequent treatments were performed at 4°C. Trypanosomes were purified from the buffy coat using PSG (NaH_2_PO_4 _3 mM, Na_2_HPO_4 _50 mM, NaCl 44 mM, sucrose 100 mM, adenosine 0.1 mM, pH 8.0). The cells were washed three times in PSG and then resuspended at 10^9 ^cells/ml.

### RNA extraction

RNA was extracted using the Qiagen RNeasy kit following the manufacturer's instructions. 1 × 10^8 ^BSF (SMB) or 5 × 10^7 ^PCF cells were used per extraction. RNA quantity and quality was measured using a NanoDrop ND-1000 (NanoDrop Technologies) and denaturing formaldehyde agarose gels [143] prior to further analysis. For the MITat1.1 cells, RNA was prepared using the Absolutely RNA kit (Stratagene) according to the manufacturer's instructions.

### Array hybridization

For each experiment, hybridisations were performed with at least three biological replicates with Cy3/Cy5 dye-swaps. Preparation of amplified and labelled cDNA targets were essentially as described [144] except that AmpliTaq DNA polymerase Buffer II (Applied Biosystems) was used for the cDNA amplification. Following hybridisation, slides were washed for 5 min in 2 × SSC, 5 min in 0.1 × SSC/0.1% SDS, 5 min in 0.1 × SSC and rinsed in dH_2_O. All washes were performed at room temperature. After washing, slides were dried by centrifugation and scanned on an Axon 4100 scanner using GenePix software (Molecular Devices). Fluorescence intensity was normalised so that the total signal for the Cy3 and the Cy5 channels were equivalent. Raw image data were extracted using Bluefuse (BlueGnome).

### Array data analysis

To identify significant differential expression, the microarray data were analysed using limma (version 2.4.7) [50] from the Bioconductor open-source project [49] running under R (version 2.2.1), and a custom program written by K. Kelly (Cambridge). Data pre-processing comprised within-array print-tip Loess normalisation and between-array scale normalisation. To identify differentially expressed mRNAs a linear model was fitted to the data, using as weights the square root of the "confidence" value given by Bluefuse for each spot (which depends on the area, circularity and uniformity of the spot, signal to noise ratio, signal saturation, and the consistency of the Cy3/Cy5 ratio in different pixels across the spot). Adjustment for multiple testing was carried out using the false discovery rate (FDR) method [145] in limma. For clustering analysis, data from the Bluefuse analysis were processed using the Gene Expression Pattern Analysis Suite (GEPAS) [146,147] using the DNMAD and SOTARRAY programs. Replicates were merged after filtering replicates with values > 0.5 – 1.0 above the median of replicates. Hierarchical clustering was performed using UPGMA and Euclidian distance with settings at normal.

### Quantitative real time (qRT)-PCR

For cDNA synthesis, 2 μg RNA was diluted to 10 μl with diethylpyrocarbonate (DEPC)-treated water and denatured at 70°C, 5 min. 15 μl of a reaction mix was added (2.5 μl dNTPs (25 mM stock), 5 μl 5× reverse transcription buffer (Invitrogen), 2 μl 100 mM DTT, 0.5 μl RNAseOUT (recombinant ribonuclease inhibitor, 40 U/μl, Invitrogen), 2 μl oligo dT (T_30_VN, 10 μM stock), 0.5 μl Superscript II Reverse Transcriptase (200 U/μl Invitrogen), and 2.5 μl DEPC-treated water) and incubated at 37°C for 1 hr, heat-inactivated at 90°C, 5 min and finally diluted to 200 μl with DEPC-treated water.

For qRT-PCR, 5 μl of cDNA was used in a 25 μl reaction including IQ SYBR Green Supermix (BioRad) with 0.4 μM gene-specific forward and reverse primers [see Additional file 2]. qRT-PCR reactions were performed in white thin-wall polypropylene multiplate 96-well unskirted PCR plates (BioRad) sealed with microseal 'B' adhesive (BioRad). Reactions were performed in a BioRad MiniOpticon real time PCR detection system and included an initial denaturation at 95°C for 3 min, 40 cycles of 95°C 30 seconds, 58°C 30 sec, 72°C 6 min (with a signal read at the end of each cycle), and a final melting curve to check fidelity from 60 – 95°C, with a signal read every 1°C. Gene-specific 20 bp primers for each gene were designed using Primer3 [148] specified to amplify a ~120 bp fragment (+/- 10 bp) in the last kilobase of the 3' end of the open reading frame and coinciding with the region recognised by the corresponding array oligonucleotide. Primer pairs were validated *in silico *using Amplify [149] to minimise the probability of mispriming or formation of primer dimers and secondary structure.

### Antibody production and Western blot analysis

TbδAd antisera was generated against an expressed gene fragment (residues 222–508) amplified from *T. brucei *genomic DNA using Herculase DNA polymerase (Stratagene) with the following primers: 5'-GGAATTCCCAGCTTCTTAGGTCTAGCGGT-3' and 5'-CCGCTCGAGCTCAACAATAGTTCGCATGTGC-3'. The 865 bp PCR product was subcloned into pPCR-Script, excised with *Eco*RI, and inserted into pGEX-2TK. Polyclonal rabbit antibodies were raised against TbδAd-GST recombinant fusion protein, which was SDS-PAGE-purified due to insolubility when expressed in *E. coli*, mixed with RIBI adjuvant (Sigma) and used to immunize rabbits on four immunisations spread over a period of five months. 0.5 mg recombinant protein was used per immunisation course.

For Western blotting, 1 × 10^7 ^trypanosome cells were harvested by centrifuging at 800 g, 10 min, 4°C, washed in phosphate-buffered saline (PBS) and resuspended in 37.5 μl PBS and 12.5 μl 4× Laemmli SDS loading buffer [143]. Lysates were denatured at 95°C for 10 min and analysed by 12.5% SDS-PAGE [143]. Equivalence of protein loading was verified by Coomassie Blue staining of a duplicate gel. Proteins were electrophoretically transferred to Immobilon-P membrane (Millipore) using a wet transfer tank (Hoefer Instruments). Nonspecific binding was blocked for 1 hr with Tris-buffered saline, pH 7.4, 0.2% Tween (TBST) supplemented with 5% freeze-dried milk. The membrane was then incubated for 1 hr with primary antibody, also diluted in TBST-milk. Rabbit polyclonal antibody against trypanosome BiP (a kind gift of James Bangs, University of Wisconsin) or the trypanosome clathrin heavy chain [16] were used at 1:1,000 dilution, and rabbit anti-TbδAd (see above) at 1:1,000. After washing 3 times for 5 min in TBST, a commercial secondary anti-IgG rabbit horse-radish-peroxidase conjugate (Sigma) was used at 1:10,000 in TBST for 1 hr, and bound-antibodies were detected on Biomax MR-1 films (Kodak) using H_2_O_2_-activated Luminol (Sigma) in 100 mM Tris pH 8.5.

## Authors' contributions

VLK and MCF designed the microarray and the experimental approach, VLK performed the microarray analysis and data interpretation, TS generated and validated the VSG RNAi line and SKAN performed immunochemical analysis of the Rab5 overexpressor lines. VLK and MCF drafted the manuscript and figures; all authors read and approved the final manuscript.

## Supplementary Material

Additional file 1 **Absence of clustering of membrane-trafficking genes in the *T. brucei *genome**. Panel A: The number of genes identified on each chromosome predicted to have a role in membrane traffic based on sequence similarity and/or domain architecture are plotted against chromosome size (in Mb). Multicopy genes are represented here as a single gene. Panel B: Raw data on which plot A is based. Approximate chromosome sizes are from GeneDB. Click here for file

Additional file 2 **Details of open reading frame selection, microarray oligonucleotides, qRT-PCR primers, database annotation and prior work**. Functional groups assigned based on likely orthologs and/or domain annotation from multiple sources. Accession numbers are for *T. brucei *927 at GeneDB, except ESAG6 and ESAG7 which are for *T. brucei *427 at GeneDB, and VSG oligonucleotides which are based on sequences deposited at NCBI. All oligonucleotide sequences are given in 5'-3' orientation. Array version 1.0 was used for the bloodstream v procyclic comparison and analysis of the Rab5 overexpressor lines; array version 1.1 used in all other experiments. The "Cross-hybridising ORFs" column gives the GeneDB accession numbers of ORFS likely to cross-hybridise to each oligonucleotide, when applicable, as predicted by the OligoArray 2.0 program. qRT-PCR oligonucleotide sequences are given for all ORFs analysed by qRT-PCR. Click here for file

Additional file 3 **Developmental regulation of components of the adaptin complexes AP1, AP3, and AP4 in *T. brucei***. Panel A: Heatmap of bloodstream versus procyclic form signal ratios for the ORFs corresponding to the adaptin complexes, based on eight microarray experiments. For each target gene, the four replicate spots on the array were averaged, following removal of inconsistent replicates. The scale shows the colour scheme for the z-score of the data, indicating how far and in what direction, the ratio for each spot deviates from the mean for each array, expressed in units of standard deviation; bright red indicates significant upregulation in BSF, bright green indicates significant upregulation in PCF, dark colours or black indicate no differential expression between the two developmental stages. AP1σ is highly upregulated in BSF, and AP3μ in PCF (also see Table 1) with a general trend of BSF upregulation for AP1 components and PCF upregulation (or BSF downregulation) for AP3 and AP4 components. Panel B: Western blot analysis of whole cell lysates from BSF (lane B) and PCF (lane P) (1 × 10^7 ^cell equivalents) probed with rabbit antisera raised against recombinant TbδAd adaptin subunit (AP3). The scale to the left represents relative molecular mass in kDa. The predicted molecular weight for the AP3δ protein is 125 kDa. Loading equivalence was monitored by Ponceau Red staining of the membrane after transfer (not shown). Click here for file

Additional file 4 **Response of *T. brucei *cells to dithiothreitol and tunicamycin treatment**. Panel A: Growth curves for BSF cultures after the addition of dithiothreitol (DTT, 1–10 mM final concentrations) or tunicamycin (5–10 μg/ml final concentrations). Cell numbers diminish rapidly after the addition of DTT (within 4 hours), whereas cell growth is arrested after addition of tunicamycin and cell numbers remain stable for up to 24 hours. Panel B: Western blot analysis of whole cell lysates (1 × 10^7 ^cell equivalents) for BiP in a control culture, as well as cultures supplemented with 1 mM DTT and 5 μg/ml tunicamycin. A culture of BSF cells was divided; one flask was retained as control and DTT or tunicamycin was added to the other subcultures. Samples were taken prior to DTT or tunicamycin addition (C0), 1 hr and 4 hr after DTT treatment (D1 and D4, respectively), 1 hr, 4 hr and 24 hr after tunicamycin treatment (T1, T4 and T24), and at 1 hr, 4 hr and 24 hr from the control (C1, C4, C24). Panel C: BiP protein levels normalised to total protein, based on the Western blot in panel B and two additional replicates. The levels of BiP antigen are normalized to 100% for the control culture at time zero (C0). Panel D: Relative levels of BiP mRNA as measured by qRT-PCR after DTT (black bars) and tunicamycin (grey bars) treatment. The levels of BiP mRNA are normalized to 100% for the control culture at time zero (C0). Click here for file

Additional file 5 **Verification of phenotypes for VSG and CLH RNAi cell lines. **Panel A: Growth curves of induced versus uninduced VSG RNAi cell lines, indicating growth arrest in the induced cell line over four days post induction. Panel B: CLH protein levels in induced (+Tet) versus uninduced (-Tet) CLH RNAi cell lines were examined by Western blotting. BiP was used as a loading control. Data from two biological replicates are shown. Click here for file

Additional file 6 **Significantly developmentally expressed trypanosome genes grouped by functional class**. Numbers of transcripts on which the graph in Figure 2 is based. The number of genes for each functional class correspond to the ORFs featured in Table 1 and discussed in the text. Note that several of the oligonucleotides on the array target multicopy genes (e.g, tubulin, histones) and thus the total number of ORFs subject to differential regulation is higher than the number of ORFs/oligonucleotides given in Table 1. Click here for file
