# Supplementary figures and images for: The trypanosome transcriptome is remodelled during differentiation but displays limited responsiveness within life stages
Source: BMC Genomics. 2008 Jun 23;9:298. doi: 10.1186/1471-2164-9-298 (PMC2443814; doi:10.1186/1471-2164-9-298)

Figure S1

A

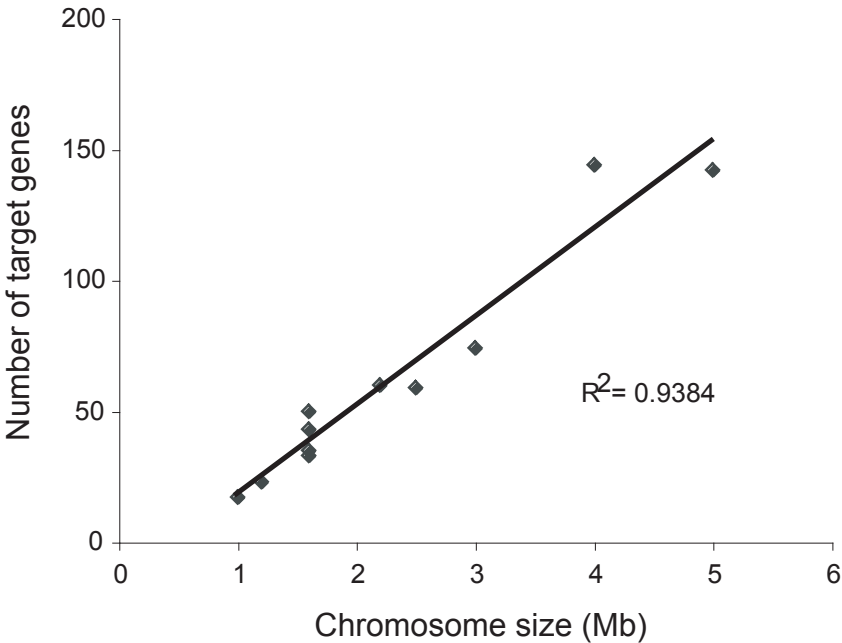

B

| Chromosome | Size (Mb) | Number of target genes |
|------------|-----------|------------------------|
| I          | 1.0       | 17                     |
| II         | 1.2       | 23                     |
| III        | 1.6       | 43                     |
| IV         | 1.6       | 50                     |
| V          | 1.6       | 33                     |
| VI         | 1.6       | 35                     |
| VII        | 2.2       | 60                     |
| VIII       | 2.5       | 59                     |
| IX         | 3.0       | 74                     |
| X          | 4.0       | 144                    |
| XI         | 5.0       | 142                    |

Supplement: Additional file 1 — Absence of clustering of membrane-trafficking genes in the T. brucei genome. Panel A: The number of genes identified on each chromosome predicted to have a role in membrane traffic based on sequence similarity and/or domain architecture are plotted against chromosome size (in Mb). Multicopy genes are represented here as a single gene. Panel B: Raw data on which plot A is based. Approximate chromosome sizes are from GeneDB. [file 1471-2164-9-298-S1.pdf]

Figure S2

A

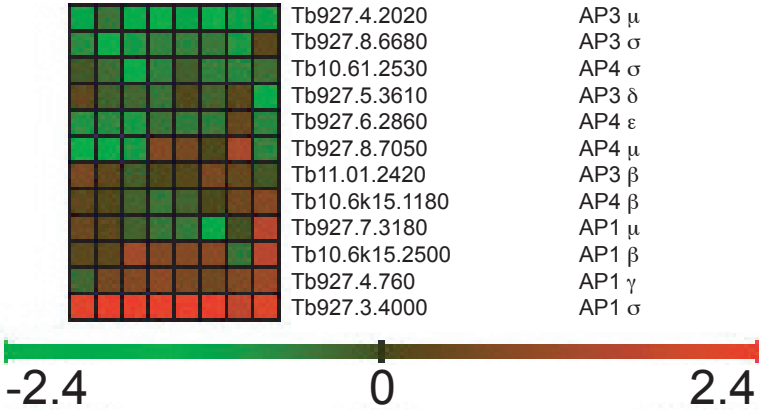

B

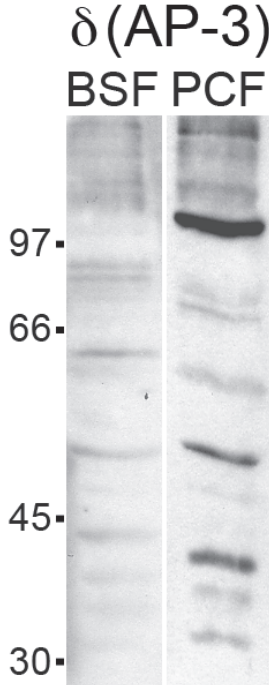

Supplement: Additional file 3 — Developmental regulation of components of the adaptin complexes AP1, AP3, and AP4 in T. brucei. Panel A: Heatmap of bloodstream versus procyclic form signal ratios for the ORFs corresponding to the adaptin complexes, based on eight microarray experiments. For each target gene, the four replicate spots on the array were averaged, following removal of inconsistent replicates. The scale shows the colour scheme for the z-score of the data, indicating how far and in what direction, the ratio for each spot deviates from the mean for each array, expressed in units of standard deviation; bright red indicates significant upregulation in BSF, bright green indicates significant upregulation in PCF, dark colours or black indicate no differential expression between the two developmental stages. AP1σ is highly upregulated in BSF, and AP3μ in PCF (also see Table 1) with a general trend of BSF upregulation for AP1 components and PCF upregulation (or BSF downregulation) for AP3 and AP4 components. Panel B: Western blot analysis of whole cell lysates from BSF (lane B) and PCF (lane P) (1 × 10^7^ cell equivalents) probed with rabbit antisera raised against recombinant TbδAd adaptin subunit (AP3). The scale to the left represents relative molecular mass in kDa. The predicted molecular weight for the AP3δ protein is 125 kDa. Loading equivalence was monitored by Ponceau Red staining of the membrane after transfer (not shown). [file 1471-2164-9-298-S3.pdf]

Figure S3

A

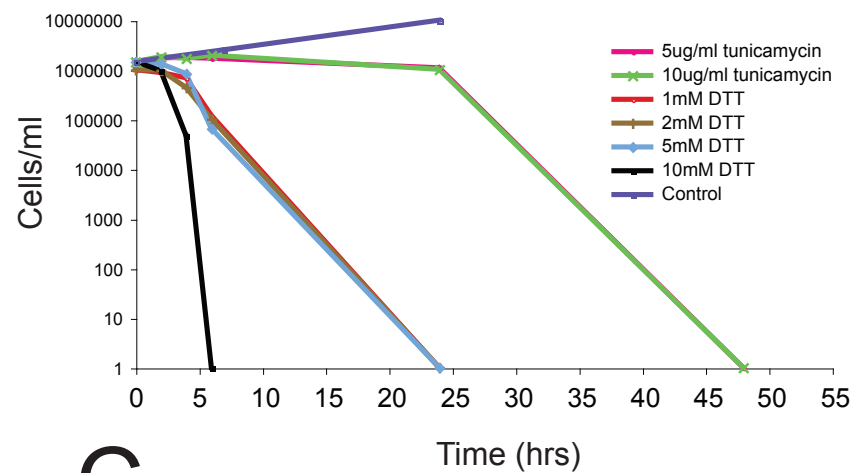

B

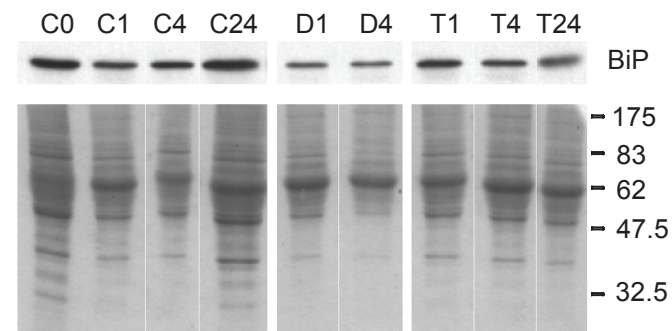

C

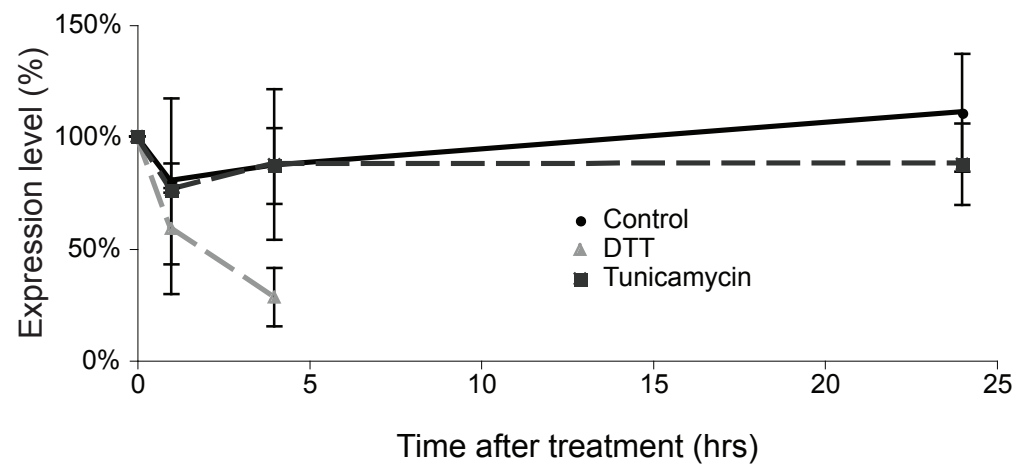

D

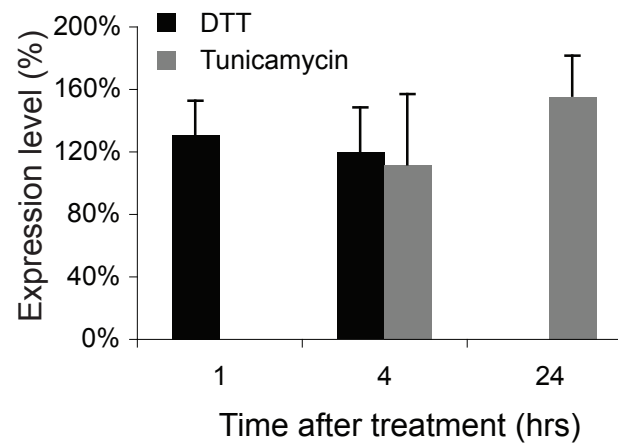

Supplement: Additional file 4 — Response of T. brucei cells to dithiothreitol and tunicamycin treatment. Panel A: Growth curves for BSF cultures after the addition of dithiothreitol (DTT, 1–10 mM final concentrations) or tunicamycin (5–10 μg/ml final concentrations). Cell numbers diminish rapidly after the addition of DTT (within 4 hours), whereas cell growth is arrested after addition of tunicamycin and cell numbers remain stable for up to 24 hours. Panel B: Western blot analysis of whole cell lysates (1 × 10^7^ cell equivalents) for BiP in a control culture, as well as cultures supplemented with 1 mM DTT and 5 μg/ml tunicamycin. A culture of BSF cells was divided; one flask was retained as control and DTT or tunicamycin was added to the other subcultures. Samples were taken prior to DTT or tunicamycin addition (C0), 1 hr and 4 hr after DTT treatment (D1 and D4, respectively), 1 hr, 4 hr and 24 hr after tunicamycin treatment (T1, T4 and T24), and at 1 hr, 4 hr and 24 hr from the control (C1, C4, C24). Panel C: BiP protein levels normalised to total protein, based on the Western blot in panel B and two additional replicates. The levels of BiP antigen are normalized to 100% for the control culture at time zero (C0). Panel D: Relative levels of BiP mRNA as measured by qRT-PCR after DTT (black bars) and tunicamycin (grey bars) treatment. The levels of BiP mRNA are normalized to 100% for the control culture at time zero (C0). [file 1471-2164-9-298-S4.pdf]

Figure S4

A

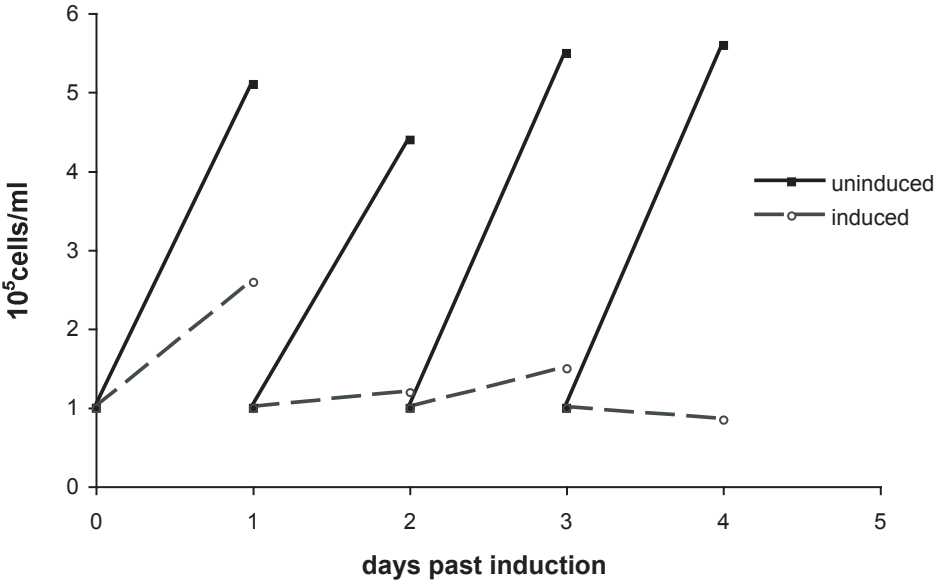

B

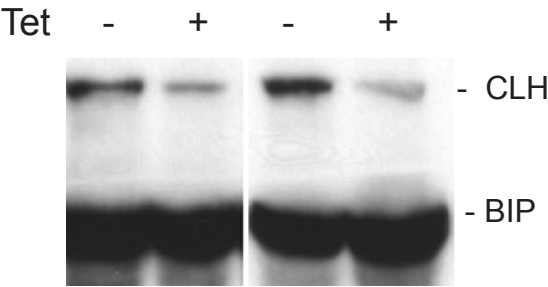

Supplement: Additional file 5 — Verification of phenotypes for VSG and CLH RNAi cell lines. Panel A: Growth curves of induced versus uninduced VSG RNAi cell lines, indicating growth arrest in the induced cell line over four days post induction. Panel B: CLH protein levels in induced (+Tet) versus uninduced (-Tet) CLH RNAi cell lines were examined by Western blotting. BiP was used as a loading control. Data from two biological replicates are shown. [file 1471-2164-9-298-S5.pdf]
