# Supplementary material for: The trypanosome transcriptome is remodelled during differentiation but displays limited responsiveness within life stages
Source: BMC Genomics. 2008 Jun 23;9:298. doi: 10.1186/1471-2164-9-298 (PMC2443814; doi:10.1186/1471-2164-9-298)
Supplement: Additional file 6 — Significantly developmentally expressed trypanosome genes grouped by functional class. Numbers of transcripts on which the graph in Figure 2 is based. The number of genes for each functional class correspond to the ORFs featured in Table 1 and discussed in the text. Note that several of the oligonucleotides on the array target multicopy genes (e.g, tubulin, histones) and thus the total number of ORFs subject to differential regulation is higher than the number of ORFs/oligonucleotides given in Table 1. [file 1471-2164-9-298-S6.pdf]

Table S2

	Total genes (version 1.0)	Increased in BSF	Increased in PCF	Equivalent
Cell surface	51	24	10	17
Chaperones	42	2	7	33
Small GTPases	61	14	0	47
G-protein regulation	48	0	0	48
Proteases	28	7	6	15
SNAREs	19	5	1	13
Tethering complexes	22	1	0	21
Vesicle coat proteins	31	5	2	24
PI kinases	25	4	2	19
Other kinases	97	6	16	75
ESCRT	13	0	1	12
Ubiquitin sorting	37	5	0	32
Other trafficking	201	15	11	175
Misc	121	8	33	80
**Total**	796	96	89	611
Percent		12	11	77
